# Dual Role of MtHAC‐1 in Regulating Cellulase and Xylanase Production in *Myceliophthora thermophila*

**DOI:** 10.1111/1751-7915.70203

**Published:** 2025-07-30

**Authors:** Yapeng Lai, Juan Wang, Ning Xie, Gang Liu, Donnabella C. Lacap‐Bugler

**Affiliations:** ^1^ Shenzhen Key Laboratory of Microbial Genetic Engineering, College of Life Sciences and Oceanography Shenzhen University Shenzhen China; ^2^ Faculty of Health and Environmental Sciences, School of Science Auckland University of Technology Auckland New Zealand

**Keywords:** cellulase, comparative transcriptomics, gene expression, Mthac‐1, *Myceliophthora thermophila*, xylanase

## Abstract

Filamentous fungi produce large quantities of cellulase and xylanase as extracellular enzymes to degrade plant‐derived polysaccharides. This process is controlled by a complex network of transcription factors (TFs). Here, we present the bZIP TF Mthac‐1 exhibiting dual regulatory effects on the production of cellulase and xylanase in *Myceliophthora thermophila*. The deletion of *Mthac‐1* reduced the cellulase and xylanase activities and protein secretion during the early phase of cultivation but enhanced in the middle and late stages of cultivation, compared with the wild‐type (WT) strain. It also led to fungal growth defects, characterised by few hyphal branching and reduced conidiation. Real‐time quantitative reverse transcription PCR (RT‐qPCR) analysis showed that Mthac‐1 dynamically regulates the expression of major cellulase genes. Furthermore, electrophoretic mobility shift assays (EMSAs) demonstrated that Mthac‐1 directly binds to the promoter regions of the β‐glucosidase gene *bgl1* (*MYCTH_66804*), cellobiohydrolase gene *cbh1* (*MYCTH_109566*), endoglucanase gene *egl2* (*MYCTH_86753*), xylanase gene *xyn1* (*MYCTH_112050*) and the regulatory gene *xyr1* (*MYCTH_2310145*), exhibiting higher binding affinity for *xyn1* and *xyr1*. The comparative transcriptomic analysis indicated that Mthac‐1 also plays an important role in the expression of 26S proteasome‐encoding genes under cellulolytic conditions. This work provides new insights into the regulatory mechanisms underlying cellulase and xylanase gene expression with potential applications in fungal strain engineering in biorefinery industries.

## Graphical abstract

Through a combination of gene deletion, overexpression analyses, and electrophoretic mobility shift assays, we demonstrate that MtHac‐1 acts as a dual‐function regulator of cellulase and xylanase production, directly modulating the expression of major cellulase and xylanase genes, as well as the regulatory gene *Mtxyr1*, through promoter binding.

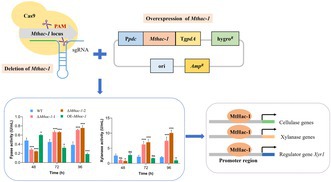

## 1 Introduction

Lignocellulosic biomass is mainly composed of cellulose (40%–50%), hemicellulose (25%–30%) and lignin (15%–20%), and represents the most abundant renewable resource on Earth that can be transformed into biofuels and value‐added biochemicals (Gupta et al. 2016). Cellulose is frequently found intermingled with xylan, the primary component of hemicellulose, to form a heterogenous complex of carbohydrate polymers (Taha et al. 2016; Xu et al. 2023). Therefore, the decomposition of xylan is considered to significantly enhance the accessibility of cellulose to cellulases, thereby boosting the overall efficiency of lignocellulose degradation (Bajaj and Mahajan 2019; Xu et al. 2023). The degradation of cellulose needs a synergistic manner of complex cellulase components, which depolymerise cellulose into glucose that can be fermented by microbes to generate biofuels such as bioethanol (Bhardwaj et al. 2021; Zhao, Liu, and Bai 2024). Like cellulose, hydrolysis of xylan requires a cooperative action of multiple xylanolytic enzymes (Bernardi et al. 2021). Generally, endo‐1,4‐β‐D‐xylanase (EC 3.2.1.8) is recognised as the key enzyme that acts on the xylan backbone to release xylo‐oligosaccharides and xylose, which possess potential industrial applications (Mendonca et al. 2023).

Filamentous fungi are the primary contributors to the depolymerisation of lignocellulose by producing an assortment of extracellular hydrolases, including cellulase and xylanase (Mattam et al. 2022). Several well‐studied fungi, such as *Trichoderma reesei*, *Penicillium oxalicum*, *Neurospora crassa* and *Myceliophthora thermophila*, have been utilised as cell factories to produce lignocellulolytic enzymes (Liu and Qu 2019; Zhang et al. 2020). Among these, the thermophilic fungus *M. thermophila* is receiving attention due to its capability to secrete large amounts of thermostable carbohydrate‐active enzymes (CAZymes) involved in the breakdown of lignocellulosic biomass (Berka et al. 2011; Singh 2016). The *M. thermophila* C1 strain has been developed as a platform to produce diverse industrially important enzymes for cost‐efficient applications (Visser et al. 2011). Recently, *M. thermophila* was genetically engineered to generate valuable enzymes and chemicals, such as cellulase, malic acid, and succinic acid, based on the multi‐omics analysis and genome‐editing technique (Liu, Zhang, et al. 2019; Li, Lin, et al. 2020; Gu et al. 2023). However, the production levels of lignocellulose‐degrading enzymes in this fungus are much lower as compared to the more commonly used mesophilic counterparts such as species of *Trichoderma* and *Aspergillus* (Berka et al. 2011).

Biosynthesis of cellulase and xylanase in filamentous fungi is regulated by a variety of transcription factors (TFs) (Benocci et al. 2017; Zhao, Zhang, et al. 2024). A few transcriptional regulators have been identified to be involved in controlling cellulase and/or xylanase gene expression in *M. thermophila* . MtXyr1 is the principal activator of genes encoding xylan‐degrading enzymes, as well as those related to pentose transport and catabolism (Dos Santos Gomes et al. 2019). Overexpression of MtXyr1 elevated xylanase production in both glucose and corncob‐containing media but showed less impact on cellulase activity (Wang et al. 2015). In contrast to MtXyr1, MtCre1 serves as an essential repressor of cellulolytic enzyme biosynthesis. RNA interference of *Mtcre1* resulted in improved cellulase production and upregulated transcript levels of cellulase genes when cultured in inducing medium (Yang et al. 2015). It was previously documented that MtClr‐4 acts as a pivotal regulator for (hemi‐) cellulase gene expression through regulating the expression of the crucial transcriptional activators MtClr‐2 and MtXyr1 (Liu, Li, et al. 2019). In addition, a Zn2Cys6 transcription factor MtClr‐5 was found to be important for cellulose degradation, as the *MtClr‐5* disruption mutant exhibited reduced protein secretion and endoglucanase activity, compared to the *M. thermophila* parental strain during growth on Avicel (microcrystalline cellulose) (Xue et al. 2023). Our recent study demonstrated that a novel forkhead protein MtFKH1 negatively regulates the major cellulase and xylanase genes via binding to their promoter regions (Lai et al. 2025). However, despite *M. thermophila* possessing a relatively higher number of xylanase‐encoding genes (Karnaouri et al. 2014), the number of identified transcription factors associated with the xylanolytic enzyme system remains lower than that in the well‐known lignocellulolytic fungus *T. reesei*. This implies a substantial potential to uncover additional regulators involved in xylan degradation. Moreover, the regulatory mechanisms of the expression of cellulolytic and xylanolytic genes in *M. thermophila* remain largely unknown (Dos Santos Gomes et al. 2019; Li et al. 2022). Understanding these regulatory mechanisms would facilitate fungal strain engineering for the hyperproduction of cellulase and xylanase in *M. thermophila* .

A special feature of filamentous fungi is the highly efficient enzyme secretion capacity, which enables them to degrade plant‐derived polymers in their natural habitat to support cellular growth (Pakula et al. 2003; Sakekar et al. 2021). During the lignocellulolytic response, newly synthesised glycoside hydrolases are sent to the endoplasmic reticulum (ER) to be folded and modified for secretion (Huberman et al. 2016). When misfolded proteins accumulate in the ER, a condition termed as ER stress activates the unfolded protein response (UPR) pathway, triggering a gene expression program that coordinates the protein‐folding machinery to mitigate the stress (Hetz and Papa 2018; Yao et al. 2023). In filamentous fungi, such as *Trichoderma reesei*, *Aspergillus nidulans*, *Aspergillus niger,* and *Neurospora crassa*, the UPR pathway is mediated by the transcription factor HacA/Hac1/Hac‐1 (Saloheimo et al. 2003; Mulder and Nikolaev 2009; Montenegro‐Montero et al. 2015). It was reported that the growth of *N. crassa* on Avicel imposed high demands on ER function (Montenegro‐Montero et al. 2015), and the lack of *hac‐1* caused a reduction in cellulase secretion capacity (Fan et al. 2015). Also, the Δ*hac‐A* strain of *Aspergillus oryzae* showed markedly downregulated expression of amylolytic enzymes in response to ER stress (Zhou et al. 2016). Additionally, the expression of *hac‐1*/*hacA* is highly induced in *N. crassa* and *A. nidulans* , respectively, during growth on cellulose (Brown et al. 2013; Fan et al. 2015). However, transcriptome profiling of *M. thermophila* cultivated in Avicel and glucose media revealed that the transcript level of *Mthac‐1* (MYCTH_2310995, a homologue of HacA/Hac‐1) was down‐regulated in response to Avicel (Lai et al. 2025), suggesting that Mthac‐1 may play a distinct role in lignocellulose deconstruction. Therefore, we decided to investigate the role of MtHac‐1 in the expression of lignocellulolytic enzyme genes of *M. thermophila* in more detail to provide the basic knowledge required for targeted strain improvement.

In this study, we investigated the function of MtHac‐1 by gene deletion and overexpression, growth phenotype analysis, electrophoretic mobility shift assays (EMSAs) and comparative transcriptomic analysis. We demonstrate that MtHac‐1 positively regulates cellulase and xylanase activity in the early stage of cultivation under cellulose but inhibits their production during the middle and late culture phases. This TF modulates the expression of major cellulase and xylanase genes, as well as the regulatory gene *Mtxyr1*, by directly binding to their promoter regions. We also show that MtHac‐1 is essential for normal hyphal development and sporulation. In addition, MtHac‐1 is involved in mediating the proteasome degradation system.

## 2 Materials and Methods

### 2.1 Strains and Culture Conditions

*Myceliophthora thermophila* (ATCC 42464) and its mutants were grown on potato dextrose agar (PDA) plates at 45°C for 7 days to produce mature conidia. For DNA extraction, mature conidia of *M. thermophila* strains were inoculated in 50 mL Mandels medium containing 2% (w/v) glucose as the carbon source as previously described (Lai et al. 2025) and cultured at 45°C for 36 h. For enzymatic activity, RT‐qPCR, and RNA‐seq analyses, the spores (approximately 5 × 10^7^) of *M. thermophila* strains were initially grown in 50 mL Mandels medium containing 2% (w/v) glucose at 45°C with shaking at 150 rpm. After 36 h of incubation, the mycelia were washed with the Mandels medium (without carbon source) and an equal amount of wet mycelia (0.55 g) was transferred to 50 mL Mandels medium with 2% (w/v) Avicel (Sigma‐Aldrich, St. Louis, MO, USA) for continued culture for 48–96 h (Lai et al. 2025). The investigation of colony morphology and conidiation on solid plates, as well as growth observation in liquid culture, were performed as previously described (Lai et al. 2025). The number of spores were counted using a haemocytometer after 7 days of incubation. The fungal mycelia in liquid culture were either used for shake‐flask study or harvested and subjected to a light microscope.

*Escherichia coli* DH5α cells were used for plasmid manipulation and propagation and were cultivated at 37°C in Luria‐Bertani (LB) medium containing ampicillin (100 μg mL^−1^).

### 2.2 Plasmid Construction

All primer sequences used in this study are listed in Table S1. For the deletion of *Mthac‐1* using the CRISPR/Cas9 system, the Cas9‐U6p‐*Mthac‐1*‐sgRNA expression cassette was constructed as previously reported (Lai et al. 2025). In brief, the *M. thermophila* U6 promoter and the protospacer sequence that fused with the sgRNA scaffold were amplified from pFC332‐Cas9‐U6p‐sgRNA scaffold vector and cloned into pFC332 vector, which generated the corresponding plasmid Cas9‐U6p‐*Mthac‐1*‐sgRNA. Approximately 1 kb of 5′ and 1.5 kb of 3′ flanking fragments of *Mthac‐1* were amplified from *M. thermophila* genomic DNA. The two resultant PCR sequences were fused with the G418‐resistance cassette P*gpd*‐*neo* amplified from plasmid pBC‐*neo* (Li, Yan, et al. 2020) and inserted into the HindIII and EcoRI sites of pUC19 plasmid using One Step Cloning Kit (Vazyme Biotech, Nanjing, China) to generate donor‐*Mthac‐1*‐*neo* vector.

For overexpression of *Mthac‐1*, the *Mthac‐1* coding region (unspliced form) was amplified from genomic DNA using the *Mthac‐1*‐F/R primer set, which was then ligated into the *Not*I and *Xba*I sites of the pUC19‐P*pdc‐*T*gpdA*‐*hph* plasmid (Lai et al. 2025), under the control of the strong constitutive *Mtpdc* (MYCTH_112121, pyruvate decarboxylase) promoter.

### 2.3 *Myceliophthora thermophila* Transformation

Protoplast preparation and transformation of *M. thermophila* was performed as previously described (Lai et al. 2025). Positive transformants were selected based on resistance to neomycin and hygromycin B (75 μg mL^−1^ hygromycin B combined with 100 μg mL^−1^ G418) or solely hygromycin B (75 μg mL^−1^) after 4–5 days of culture. Successful transformants were further confirmed through genomic PCR analysis and sequencing using different specific primer pairs (Table S1).

### 2.4 Total DNA and RNA Extraction

Total fungal DNA and RNA were extracted from fresh mycelia collected from corresponding liquid culture via vacuum filtration. The harvested mycelia were mechanically ground into fine powder in liquid nitrogen for subsequent use. The DNA and RNA were extracted using the fungal DNA extraction kit (Sangon Biotech, Shanghai, China) and RNA extraction kit TransZol Up (TransGen Biotech, Beijing, China), respectively, according to the manufacturers' instructions. Agarose gel electrophoresis and a NanoDrop 2000 Spectrophotometer (Thermo Scientific, Waltham, MA, USA) were used to evaluate the quality and quantity of the DNA and RNA.

### 2.5 Real‐Time Quantitative PCR

The HiScript III RT SuperMix kit (Vazyme Biotech, Nanjing, China) was employed to synthesise cDNA from RNA following the manufacturer's protocols. Each PCR was performed in a 20 μL reaction mixture containing 10 μL of Hieff UNICON Universal Blue qPCR SYBR Green Master Mix (Yeasen Biotech, Shanghai, China), 0.4 μL of 10 μM forward primer, 0.4 μL of 10 μM reverse primer (Table S1), 2.0 μL of diluted cDNA and 7.2 μL of sterile water. The thermocycler condition was as follows: initial denaturation for 2 min at 95°C, followed by 40 cycles of 5 s at 95°C and 20 s at 63°C. The relative expression level of each gene was calculated by the comparative 2^−ΔΔ*CT*^ method (Livak and Schmittgen 2001) with the *actin* gene (*MYCTH_2314852*) of *M. thermophila* as an endogenous control.

### 2.6 Transcriptome Analysis

The total RNA isolated from the wild type and Δ*Mthac‐1* strains of *M. thermophila* cultured on 2% (w/v) Avicel for 48 h after shift from glucose was utilised for RNA‐seq on the Illumina Novaseq6000 platform by Gene Denovo Biotechnology Corporation (Guangzhou, China). The generated high‐quality reads were mapped to the genome sequence of *M. thermophila* ATCC 42464 using HISAT2. v2.4 (Kim et al. 2015). The expression abundance (FPKM, fragment per kilobase of transcript per million mapped reads) of each gene was calculated by the RSEM software (Li and Dewey 2011). DESeq2 was employed for analysis of differential gene expression with thresholds of absolute fold change > 2 and adjusted *p*‐value (*p*_adj_) < 0.05 (Love et al. 2014). Gene Ontology (GO) enrichment assay was performed using the OmicShare tools (https://www.omicshare.com/tools). All differentially expressed genes (DEGs) were mapped against GO terms in the Gene Ontology database (http://www.geneontology.org/). GO terms with *p*‐value < 0.05 were considered significantly enriched compared with the *M. thermophila* genome background using hypergeometric test. The raw reads of transcriptome data have been deposited in the Gene Expression Omnibus (accession number: GSE290641) in the National Center for Biotechnology Information (NCBI).

### 2.7 Protein and Enzyme Activity Assays

Samples of crude enzyme were collected at the indicated time for the analyses of extracellular enzyme activity. The protein concentration in culture supernatant was measured using a Modified Bradford Protein Assay Kit (Sangon Biotech, Shanghai, China) with bovine serum albumin (BSA) as the standard. The absorbance of the reaction mixture was determined at 595 nm. For sodium dodecyl sulfate‐polyacrylamide gel electrophoresis (SDS‐PAGE) analysis, 20 μL of unconcentrated culture supernatant was subjected to a 10% polyacrylamide gel with the aid of the PAGE Gel Quick Preparation Kit (Yeasen Biotech, Nanjing, China). Cellulase activities including filter paper cellulase (FPase), endoglucanase (CMCase), β‐glucosidase (pNPGase) and exoglucanase (pNPCase) activities, as well as xylanase activity were measured as previously described (Lai et al. 2025). One unit (U) for FPase, CMCase, and xylanase activity was defined as the amount of 1 μmol glucose or 1 μmol xylose produced by 1 mL enzyme from the substrate per minute under standard assay conditions. One unit (U) for pNPGase and pNPCase activity was the amount of 1 μmol *p*‐nitrophenol (pNP) released by 1 mL enzyme per minute from the appropriate substrate. Triplicate independent biological experiments were conducted for each sample.

### 2.8 Electrophoretic Mobility Shift Assays

The DNA sequence encoding the putative DNA‐binding domain MtHAC‐1_126–190_ was amplified from *M. thermophila* genomic DNA using the primer set shown in Table S1. The purified PCR fragments were ligated into the *HindIII* site of pET‐51b to form a Strep II‐tagged protein expression plasmid, which was then introduced into *E. coli* BL21(DE3) for protein expression. The expression and purification of recombinant protein were performed as previously reported (Lai et al. 2025).

EMSAs were conducted as described previously (Wang et al. 2012; Lai et al. 2025). In brief, promoter regions of *bgl1* (MYCTH_66804) (P2, −650 to −300), *cbh1* (MYCTH_109566) (P1, −350 to −1), *egl2* (MYCTH_86753) (P2, −650 to −300), *xyn1* (MYCTH_112050) (P2, −650 to −300; P3, −1000 to −650), and *xyr1* (MYCTH_2310145) (P2, −650 to −300) were amplified from *M. thermophila* genomic DNA using specific primers (Table S1) and used as probes. In each EMSA reaction, various amounts (0–0.8 μg) of MtHAC‐1_126–190_ were incubated with a constant quantity (100 ng) of the DNA probes at 25°C for 30 min in binding buffer. Purified Strep II‐tagged protein from *E. coli* BL21(DE3) cells harboured with the vector pET‐51b‐MtCLR‐2_30–89_ was used as the negative control.

## 3 Results

### 3.1 Identification of MtHAC‐1 as a bZIP‐Type Protein

**FIGURE 1:**
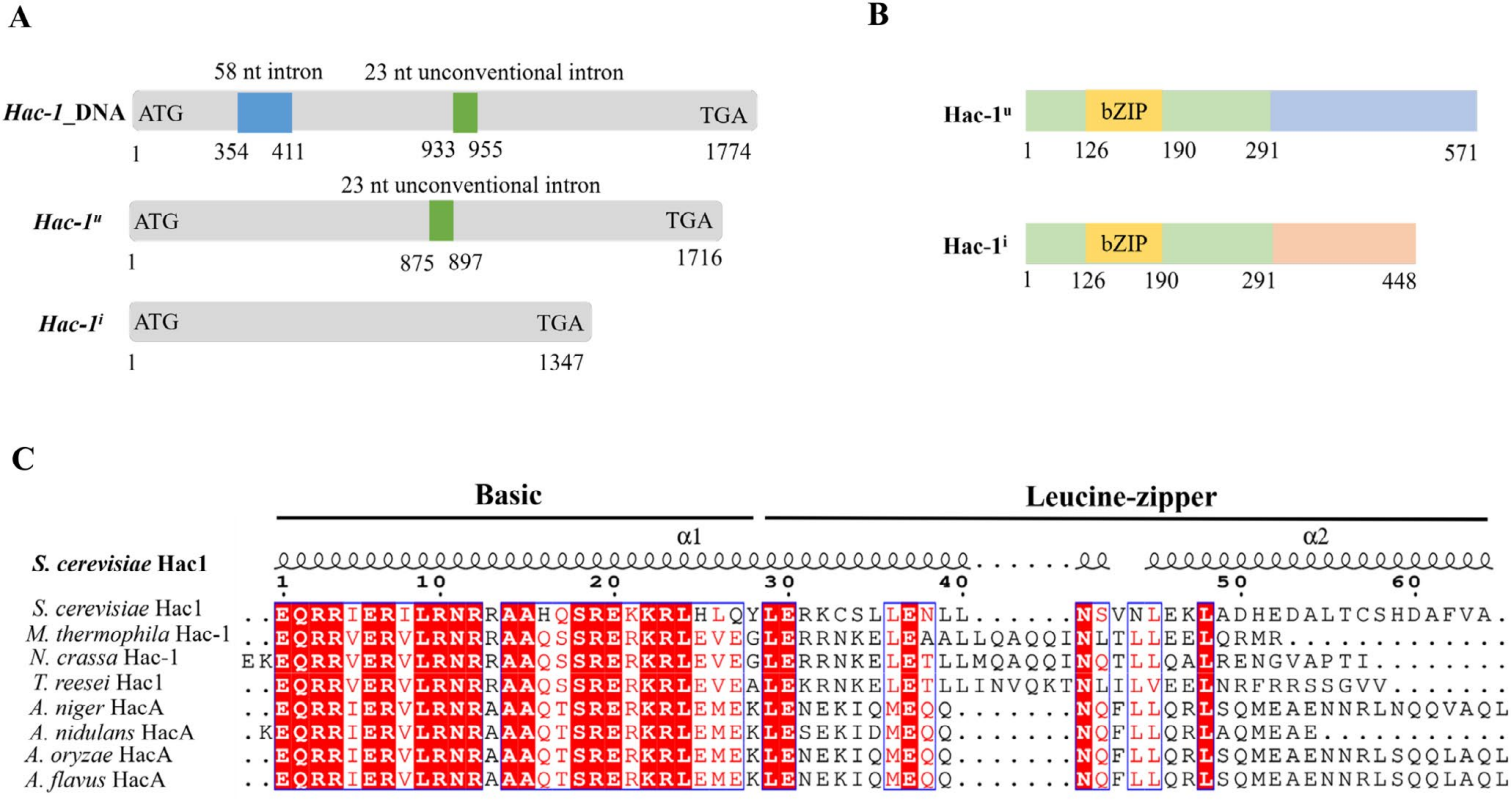
MtHAC‐1 is a putative bZIP‐type protein. (A) The gene structure of *Mthac‐1* DNA and mRNA. (B) Schematic representation of Mthac‐1 amino acids. Yellow box, bZIP domain; Blue box, unique C‐terminus of MtHac‐1^u^; Orange box, unique C‐terminus of MtHac‐1^i^; Green box, shared N‐terminus of both MtHac‐1^u^ and MtHac‐1^i^. (C) Alignment of protein sequences of the conserved bZIP domain in selected fungal MtHAC‐1 homologues. The α represents α‐helix, which is based on *S. cerevisiae* Hac1, obtained from SWISS‐MODEL. The identical amino acids are presented as white letters in a red background, and amino acids in red letters within blue frames indicate similar physical and chemical properties. *Hac‐1* ^*u*^, unspliced form of *Mthac‐1* mRNA; *Hac‐1* ^*i*^, noncanonical spliced form of *Mthac‐1* mRNA.

The *M. thermophila Mthac‐1* gene (MYCTH_2310995) is 1774 bp in length and contains a traditional intron of 58 nt, the removal of which creates the *Mthac‐1* mRNA of 1716 nucleotides (Figure 1A). A previous study demonstrated that the growth of *M. thermophila* in cellulose‐containing medium induces endoplasmic reticulum (ER) stress, resulting in the unconventional splicing of a 23‐nt intron from the *Mthac‐1* mRNA, thereby producing a mature transcript of 1347 nucleotides (Figure 1A) (Li et al. 2022). The MtHac‐1 protein consists of 571 amino acids and contains a typical basic leucine‐zipper (bZIP) domain (Figure 1B). However, the removal of the 23‐nt intron would alter the open reading frame of the *M. thermophila hac‐1* mRNA, resulting in the production of a 448‐amino‐acid protein that shares an identical N‐terminus but has a distinct C‐terminus compared to the conventional MtHAC‐1 protein (Figure 1B) (Li et al. 2022). The bZIP domain is a contiguous α‐helix that is composed of a basic region mediating DNA binding and a leucine zipper motif needed for dimerisation (Maldonado‐Bonilla 2020). The Hac‐1 homologues from *Saccharomyces cerevisiae* , *N. crassa* , *T. reesei*, *A. niger* , *A. nidulans* , *A. oryzae,* and *Aspergillus flavus* have been characterised (Kaufman 1999; Saloheimo et al. 2003; Mulder and Nikolaev 2009; Montenegro‐Montero et al. 2015). The alignment of these proteins from different fungal species with MtHac‐1 revealed that the basic motif is highly conserved, whereas the leucine zipper is more variable (Figure 1C).

### 3.2 Dual Effects of Mthac‐1 on Cellulase and Xylanase Induction in *M. thermophila*

**FIGURE 2:**
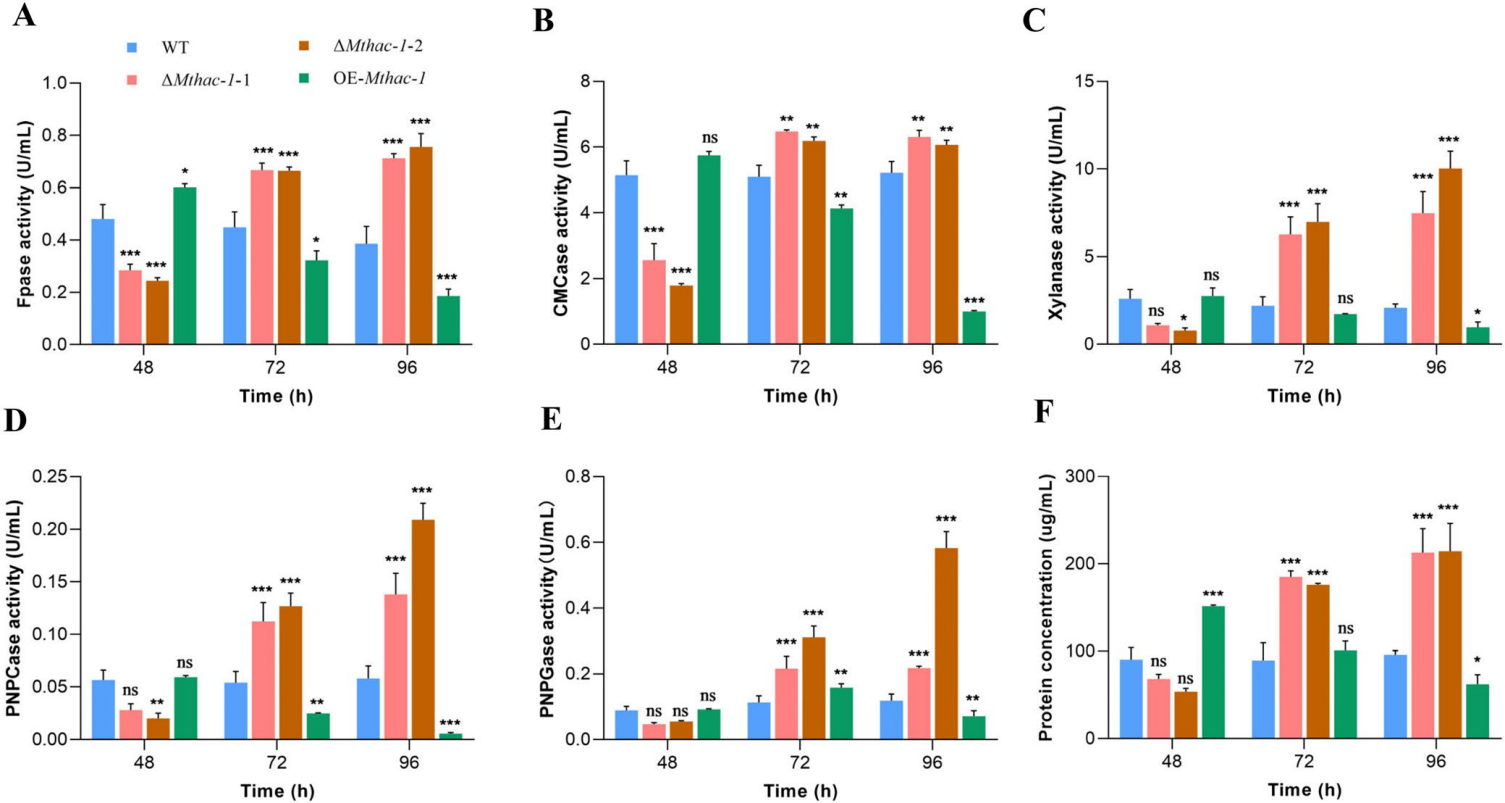
Cellulase and xylanase activities (A–E), and total protein (F) in supernatants from cultures of *M. thermophila* WT strain, Δ*Mthac‐1* mutants and OE‐*Mthac‐1* strain. (A) Filter paper cellulase (FPase) activity. (B) Endoglucanase (CMCase) activity. (C) Xylanase activity. (D) Cellobiohydrolase (pNPCase) activity. (E) β‐glucosidase (pNPGase) activity. (F) Extracellular protein production. These *M. thermophila* strains were grown on glucose for 36 h and subsequently transferred to 2% Avicel medium and cultivated for 48–96 h. *p < 0.05, **p < 0.01 and ***p < 0.001 indicate significant differences between the WT and Δ*Mthac‐1* mutants or OE‐*Mthac‐1* strain (two‐way ANOVA), ns indicates not significant. Error bars represent the SD from three replicates. ∆*Mthac‐1*‐1 and ∆*Mthac‐1*‐2, represent two independently constructed ∆*Mthac‐1* mutant strains.

To investigate the role of Mthac‐1 in cellulase and xylanase production, the *Mthac‐1* deletion mutants were generated by using the CRISPR/Cas9 system (Figure S1). The Cas9‐U6p‐sgRNA expression cassette targeting *Mthac‐1* was co‐transformed into *M. thermophila* wild‐type (WT) protoplasts along with a donor DNA vector containing the P*gpd‐neo* selection marker and the 5′ and 3′ homologous arms of *Mthac‐1*. Deletion of the *Mthac‐1* gene was accomplished through homology‐directed repair (HDR) mediated by the CRISPR/Cas9 system. Finally, two independent ∆*Mthac‐1* mutants, designated as ∆*Mthac‐1*‐1 and ∆*Mthac‐1*‐2, were successfully obtained and subsequently used for further analysis. Moreover, the encoding sequence of *Mthac‐1* driven by the strong constitutive promoter P*pdc* (pyruvate decarboxylase, MYCTH_112121) was integrated ectopically into the genome of *M. thermophila* wild‐type (WT) to create the *Mthac‐1*‐overexpression (OE‐*Mthac‐1*) strain. The correct deletion and integration events in the obtained mutants were verified by genomic PCR with several specific primers (Table S1, Figure S2). After growth in Avicel medium for 48 h, the FPase, CMCase, xylanase, pNPCase and pNPGase activities, and secreted protein of *Mthac‐1* deletion mutants decreased by 41% to 49%, 50% to 65%, 58% to 70%, 51% to 63%, 38% to 47%, and 25% to 41% respectively, compared with those of the WT (Figure 2A–F). However, compared to the WT, Δ*Mthac‐1* strains showed a significant elevation in FPase activity (49% to 100%), CMCase activity (16% to 27%), xylanase activity (185% to 440%), pNPCase activity (107% to 260%), and pNPGase activity (58% to 390%) after cultivation in Avicel medium for 72 and 96 h (Figure 2A–E). Additionally, Δ*Mthac‐1* mutants secreted 97% to 124% higher protein than WT on Avicel at 72 and 96 h (Figure 2F), which was confirmed by extracellular secretome profiling through SDS‐PAGE (Figure S3). In contrast to Δ*Mthac‐1* mutants, improved FPase activity (25%) and extracellular protein production (68%) were observed in the OE‐*Mthac‐1* strain when grown on Avicel for 48 h, although a low increase in the CMCase activity (12%) was also detected (Figure 2A,B,F). As expected, after 72 and 96 h of cultivation, the OE‐*Mthac‐1* strain exhibited a noticeable reduction in FPase activity (28% to 52%), CMCase activity (19% to 81%), xylanase activity (22% to 47%), and pNPCase activity (54% to 90%), in comparison to those of the WT strain (Figure 2A–D). Furthermore, while the pNPGase activity and secreted protein of the OE‐*Mthac‐1* strain increased by 40% and 12%, respectively, relative to the WT during the presence of Avicel at 72 h, their production decreased by 40% and 35%, correspondingly, after 96 h of growth (Figure 2E,F). Notably, although ∆*Mthac‐1*‐1 and ∆*Mthac‐1*‐2 exhibited minor differences in enzymatic activities, both strains displayed consistent phenotypic trends, supporting the reliability and robustness of the ∆*Mthac‐1* phenotype. Such variation is expected and may result from differences in homologous recombination events mediated by the CRISPR/Cas9 system (Huang and Cook 2022). Taken together, results indicate that Mthac‐1 plays a positive role in regulating cellulase and xylanase activities during the early phase of growth on Avicel but acts as a repressor in the middle and later stages.

### 3.3 The *Mthac‐1* Gene Is Required for Normal Radial Growth and Conidiation on Solid Media

**FIGURE 3:**
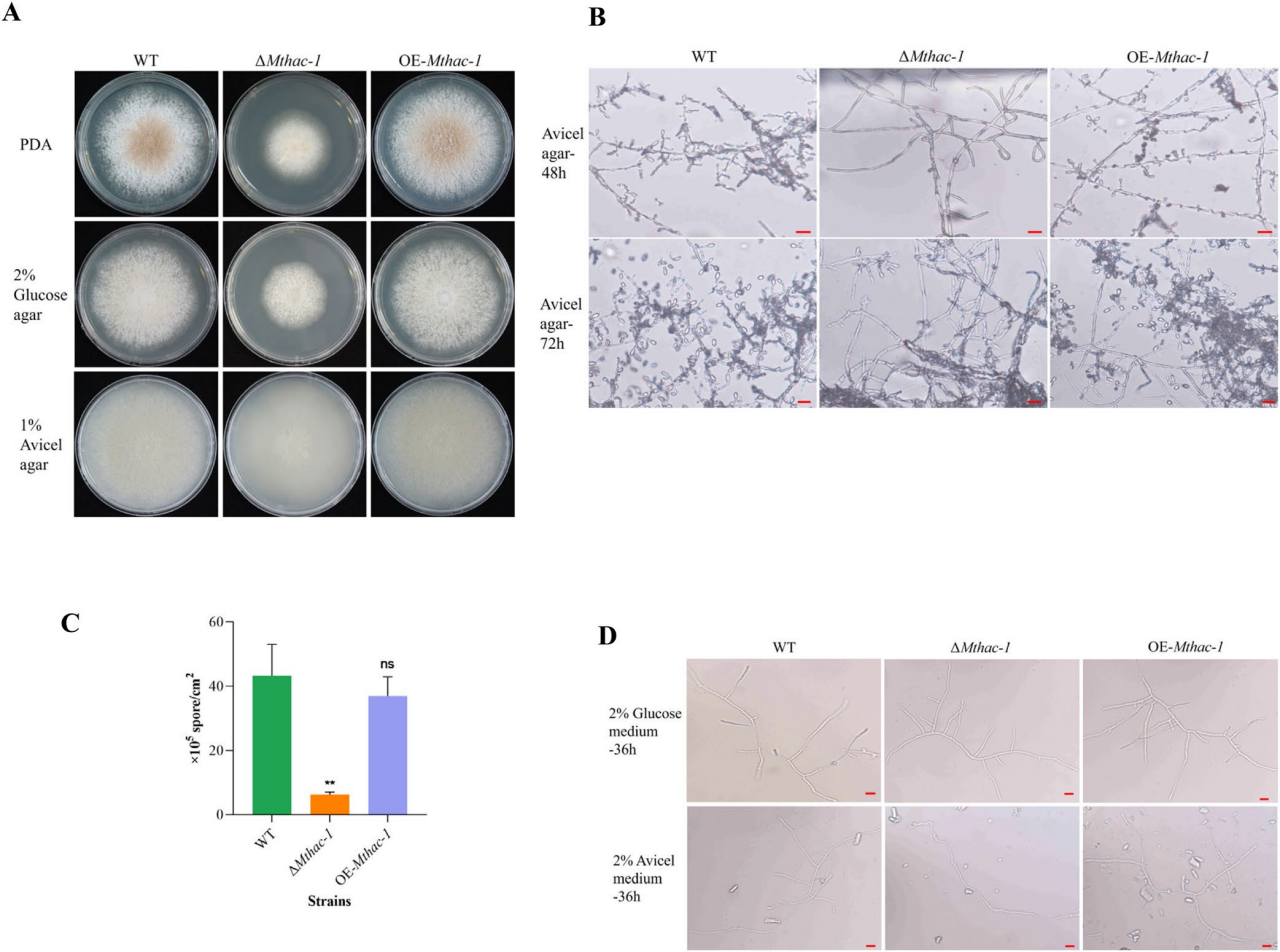
Growth phenotype analyses of *M. thermophila* WT, Δ*Mthac‐1* and OE‐*Mthac‐1* strain*s. (*A) Fungal colonies on PDA, agar plates containing 2% (w/v) glucose or 1% (w/v) Avicel for 3 (PDA) or 4 days (glucose and Avicel) at 45°C. (B) Microscopic investigation of fungal hypha and conidiation. Fungal strains were grown on 1% (w/v) solid Avicel medium for 48 and 72 h. Scale bar = 10 μm. (C) Quantitative determination of fungal sporulation when cultured in 1% (w/v) solid Avicel medium for 7 days at 45°C. **p < 0.01 represents a significant difference in conidiation between WT and Δ*Mthac‐1* strains (Student's *t*‐tests), ns indicates no significant difference between WT and OE*‐Mthac*‐*1* strains. Error bars indicate the SD from three replicates. (D) Microscopic observation of fungal mycelia grown in liquid glucose or Avicel media at 45°C for 36 h. Scale bar = 10 μm.

To investigate the effects of MtHac‐1 on the growth of *M. thermophila* , we compared the colony phenotypes of the WT, Δ*Mthac‐1* mutant and OE‐*Mthac‐1* strains on PDA medium and solid agar plates containing glucose or Avicel. The Δ*Mthac‐1* mutant formed smaller and more compact colonies in comparison to the WT on all tested carbon sources (Figure 3A). As shown in Figure 3B, after 48 h of inoculation, the Δ*Mthac‐1* mutant developed few hyphae branching with no developing conidia, compared with the normal conidiogenesis of the WT. After 72 h of incubation, the *Mthac‐1* deletion strain produced only a few conidia (Figure 3B). This observation was quantitatively confirmed, as the Δ*Mthac‐1* strain generated only 15% of the spores produced by the WT when cultivated on Avicel for 7 days (Figure 3C).

Given the impairment in radial growth, we further examined the mycelial growth of the Δ*Mthac‐1* mutant, along with the WT and OE‐*Mthac‐1* strains, in liquid shake flasks containing glucose or Avicel. The results showed that the growth of the *Mthac‐1* disruption mutant on Avicel was severely impaired at 36 h (Figure 3D). However, the Δ*Mthac‐1* strain gradually developed normal mycelia at 72 and 96 h, similar to that formed by the WT after 36 h (Data not shown). This was consistent with the extracellular protein production pattern observed in the *Mthac‐1* deletion mutant (Figure 2F). To utilise lignocellulose, filamentous fungi need to secrete numerous enzymes to degrade this complex polymer into monosaccharides and/or oligosaccharides, which can then be absorbed by the cells to support growth (Benocci et al. 2017; Gu et al. 2023). Since efficient cellulase production is vital for mycelial growth on cellulose, we reasoned that the growth defects of the Δ*Mthac‐1* mutant in liquid Avicel culture during the early phase may be due to a reduced cellulase secretion capacity on this carbon source. Interestingly, growth on glucose was unaffected in the Δ*Mthac‐1* strain (Figure 3D), indicating a specific defect correlated with cellulose metabolism, which supports our hypothesis. This is similar to *N. crassa* , where the growth of the corresponding mutant was impaired under liquid cellulose culture but was comparable to that of the WT in liquid media containing sucrose or xylan (Montenegro‐Montero et al. 2015). Correspondingly, microscopic examination revealed that the Δ*Mthac‐1* strain developed aberrant mycelia with no hyphal branching after 36 h of growth in liquid Avicel medium. In contrast, it exhibited vegetative mycelial growth comparable to the WT when grown in liquid glucose culture (Figure 3D). Notably, the OE‐*Mthac‐1* strain displayed indistinguishable growth from the WT on media containing different carbon sources, both under solid and liquid culture conditions (Figure 3A–D). Collectively, these findings suggest that MtHac‐1 is essential for normal radial growth, conidiation on solid media and mycelial development in liquid cellulose culture during the early stage.

### 3.4 MtHac‐1 Regulates the Expression of Major Cellulase and Xylanase Genes and the Regulatory Gene *xyr1*

**FIGURE 4:**
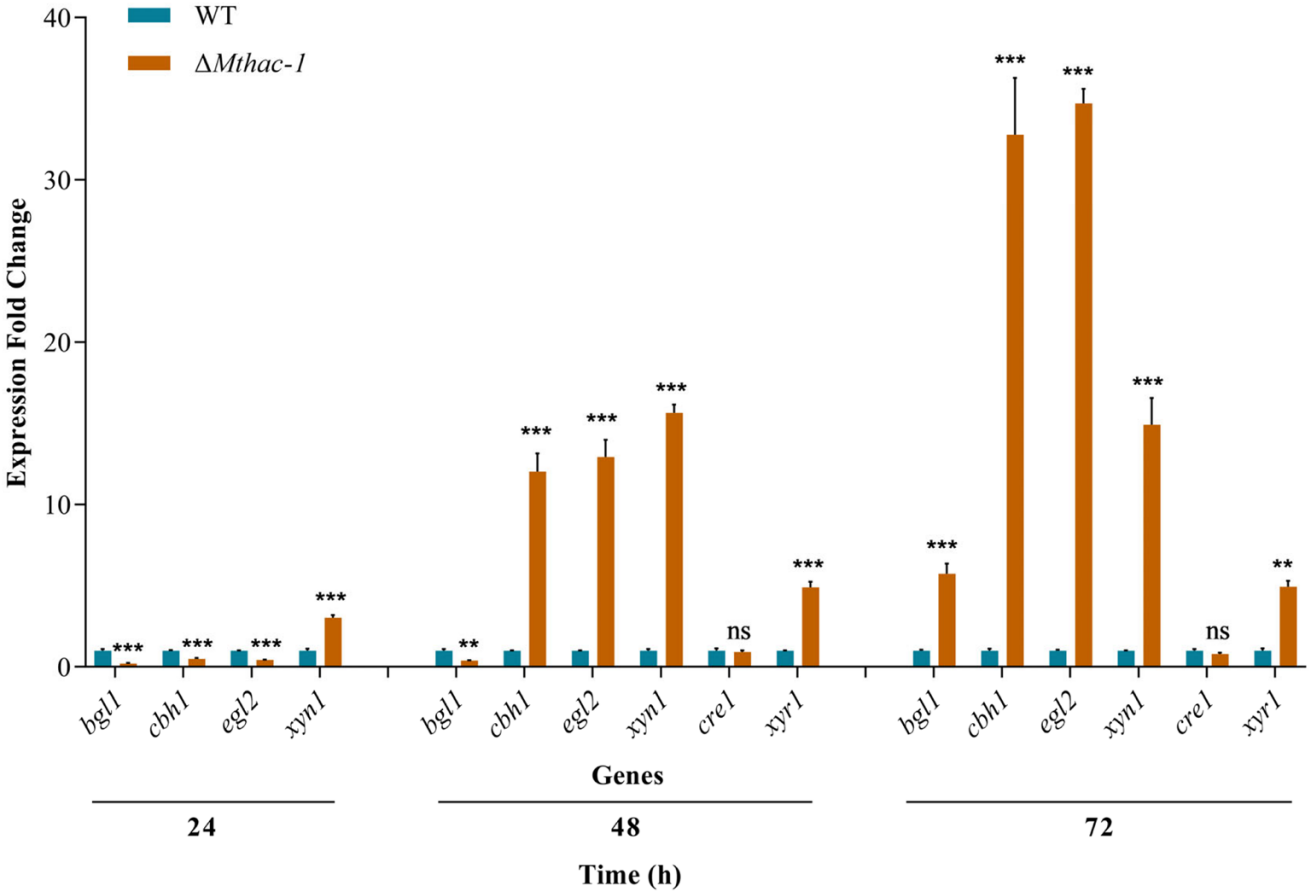
The transcription levels of major cellulase and xylanase genes and the regulatory genes *cre1* and *xyr1* in the *M. thermophila* Δ*Mthac‐1* mutant compared to the WT strain. Strains were pre‐grown in glucose for 36 h, washed and transferred to 2% (w/v) Avicel medium and cultivated for 24, 48, and 72 h. The transcript level of each gene in Δ*Mthac‐1* was normalised to the level of the corresponding gene in WT. **p < 0.01 and ***p < 0.001 represent significant differences between the Δ*Mthac‐1* and WT strains (two‐way ANOVA), ns indicate no significant difference. Error bars represent the SD from three replicates.

The findings suggest that MtHac‐1 is involved in regulating cellulase and xylanase production in *M. thermophila* , possibly by modulating cellulase and xylanase genes and/or vital transcription factor genes at the mRNA level. To investigate this further, RT‐qPCR was employed to analyse the expression of the β‐glucosidase gene *bgl1* (*MYCTH_66804*), cellobiohydrolase gene *cbh1* (*MYCTH_109566*), endoglucanase gene *egl2* (*MYCTH_86753*), xylanase gene *xyn1* (*MYCTH_112050*), and the regulatory genes including *cre1* (*MYCTH_2310085*) and *xyr1* (*MYCTH_2310145*). Expression levels were measured in *M. thermophila* WT and Δ*Mthac‐1* strains grown in Avicel medium for 24, 48, and 72 h following transfer from glucose. The transcript levels of *cbh1* and *egl2* decreased by 50% to 56% in Δ*Mthac‐1* compared with the WT after 24 h of growth on Avicel; however, their expression exhibited 12.0‐ to 34.7‐fold elevations at 48 and 72 h (Figure 4). Similarly, *bgl1* expression was reduced by 61% to 80% in the *Mthac‐1* disruption strain at 24 and 48 h but displayed a 4.8‐fold enhancement at 72 h (Figure 4). In addition, *xyn1* showed consistently elevated expression ranging from 3.0‐ to 15.7‐fold in Δ*Mthac‐1* compared with the WT during the entire cultivation period (24–72 h) (Figure 4). Furthermore, the transcript levels of *xyr1* were substantially increased (approximately 4.0‐fold) at 48 and 72 h in strain Δ*Mthac‐1*, whereas *cre1* expression remained unchanged (Figure 4). These expression patterns, except for the abnormal upregulation of *xyn1* at 24 h, agreed with the enhanced protein secretion and enzymatic activities observed in the Δ*Mthac‐1* strain during the presence of Avicel.

### 3.5 MtHAC‐1 Can Bind to the Promoter Regions of Major Cellulase and Xylanase Genes and the Regulatory Gene *xyr1*

**FIGURE 5:**
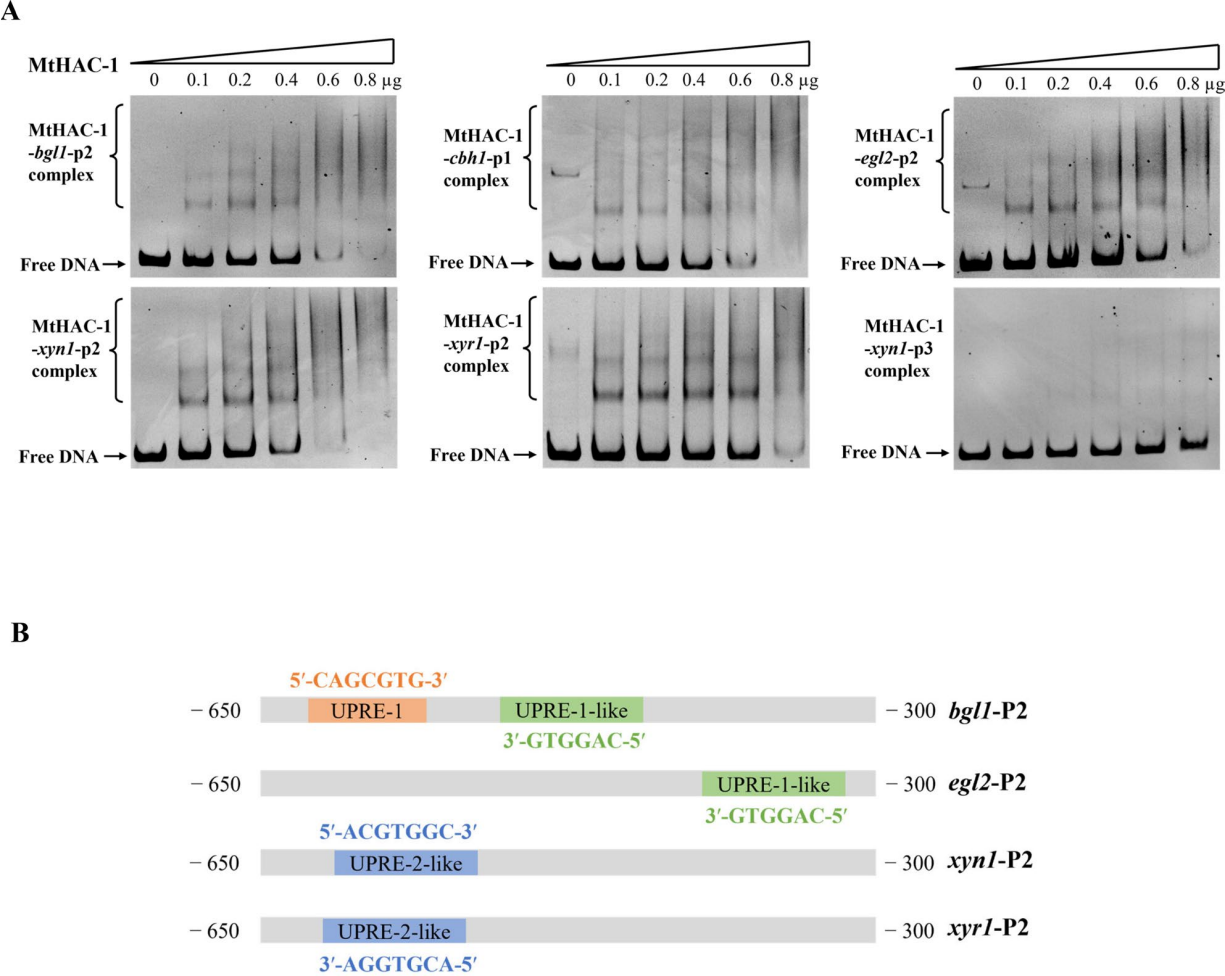
Identification of MtHAC‐1 binding motifs in the promoter regions of target genes. (A) Electrophoretic mobility shift assays (EMSA) of the binding between MtHAC‐1 and the promoter regions of genes encoding cellulase and xylanase, and regulatory gene *xyr1*. Each reaction system contains 100 ng of candidate probe and indicated amounts of purified MtHAC‐1 binding domain. The promoter of *xyn1*(*xyn1*‐p3) was employed as negative control. (B) Schematic demonstration of the putative MtHAC‐1binding motifs in the promoter regions of experimentally confirmed target genes (*bgl1*, *egl2*, *xyn1,o* and *xyr1*). Box in orange indicates UPRE‐1 motif, boxes in green represent UPRE‐1‐like motif, while boxes in blue stand for UPRE‐2‐like motif.

The RT‐qPCR data revealed that MtHAC‐1 controls the transcription levels of key cellulase and xylanase genes and the crucial regulatory gene *xyr1*. To confirm whether MtHAC‐1 directly or indirectly regulates the expression of these target genes, electrophoretic mobility shift assays (EMSAs) were conducted, involving the DNA‐binding domain of MtHAC‐1 and the promoter regions of targeted genes. It is generally challenging to accomplish recombinant expression of full‐length regulatory proteins in *E. coli* . Therefore, MtHAC‐1_126–190_, containing the putative bZIP domain, was expressed in *E. coli* BL21(DE3) and purified to generate the fusion protein MtHAC‐1_126–190_ with Strep II‐His tag (Figure S4). Probes covering the promoter regions of *bgl1* (P2, −650 to −300), *cbh1* (P1, −350 to −1), *egl2* (P2, −650 to −300), *xyn1* (P2, −650 to −300; P3, −1000 to −650), and *xyr1* (P2, −650 to −300) were amplified by PCR using specific primer pairs. In the EMSAs, the recombinant MtHAC‐1_126–190_ bound to the promoter regions of both *bgl1*, *cbh1*, *egl2*, *xyn1,* and *xyr1* in a protein concentration‐dependent pattern (Figure 5A). The binding complex bands occurred upon the addition of 0.1 μg MtHAC‐1_126–190_. This binding was specific since no retardation was observed in the promoter of *xyn1*‐P3 (Figure 5A). Additionally, MtHAC‐1_126–190_ displayed a high affinity for the probe fragments of the *xyn1*‐P2 and *xyr1*‐P2 promoter regions, while weak binding capacity was detected with the *bgl1*, *cbh1,* and *egl2* promoter regions (Figure 5A). Purified Strep II‐fused MtCLR‐2_30–89_ was used as a negative control to exclude nonspecific binding (Data not shown). These findings illustrate that MtHAC‐1 regulates cellulase and xylanase gene expression through two pathways: direct binding and through essential TF gene *Mtxyr1*.

In *S. cerevisiae* , 5′‐CAGCGTG‐3′, a partly palindromic sequence (underlined) around a spacer of one nucleotide, which is termed the UPRE‐1 (unfolded protein response element), was demonstrated to be essential for Hac1 binding (Mori et al. 1998). Subsequent studies revealed that a large set of Hac1‐regulated genes, although lacking the UPRE‐1 sequence, contain the UPRE‐2 motif (5′‐TACGTG‐3′) (Patil et al. 2004). In addition, bioinformatic analysis found that UPRE‐2‐like motif (5′‐ACGTG(T/G)(C/A)‐3′) exhibited strong binding interactions with ScHac1 (Fordyce et al. 2012). To determine the MtHAC‐1 binding sequence in the promoter regions of target genes, these identified ScHac1‐binding motifs were used to search the corresponding promoter regions of *bgl1*, *cbh1*, *egl2*, *xyn1* and *xyr1*. The results identified binding sites that are consistent with these conventional sequences in the upstream regions of *bgl1* (UPRE‐1), *xyn1* (UPRE‐2‐like) and *xyr1* (UPRE‐2‐like) (Figure 5B). This is in line with the observation in *A. niger* , where the sequence 5′‐ACACGTGTCCT‐3′ resembles the UPRE‐2‐like motif (underlined), exhibiting much stronger binding capacity with HacA (Mulder et al. 2006). Moreover, one similar binding motif was also detected in the promoter regions of *bgl1* and *egl2*, which was referred to as the UPRE‐1‐like motif (Figure 5B). However, no sequences matching any known binding consensus were found in the *cbh1* promoter, indicating that additional, yet unrecognised, oligonucleotide sequences may be required for the binding of MtHAC‐1 to genes lacking canonical motifs.

### 3.6 Transcriptomic Analysis of the *Mthac‐1* Deletion Mutant on Avicel

**FIGURE 6:**
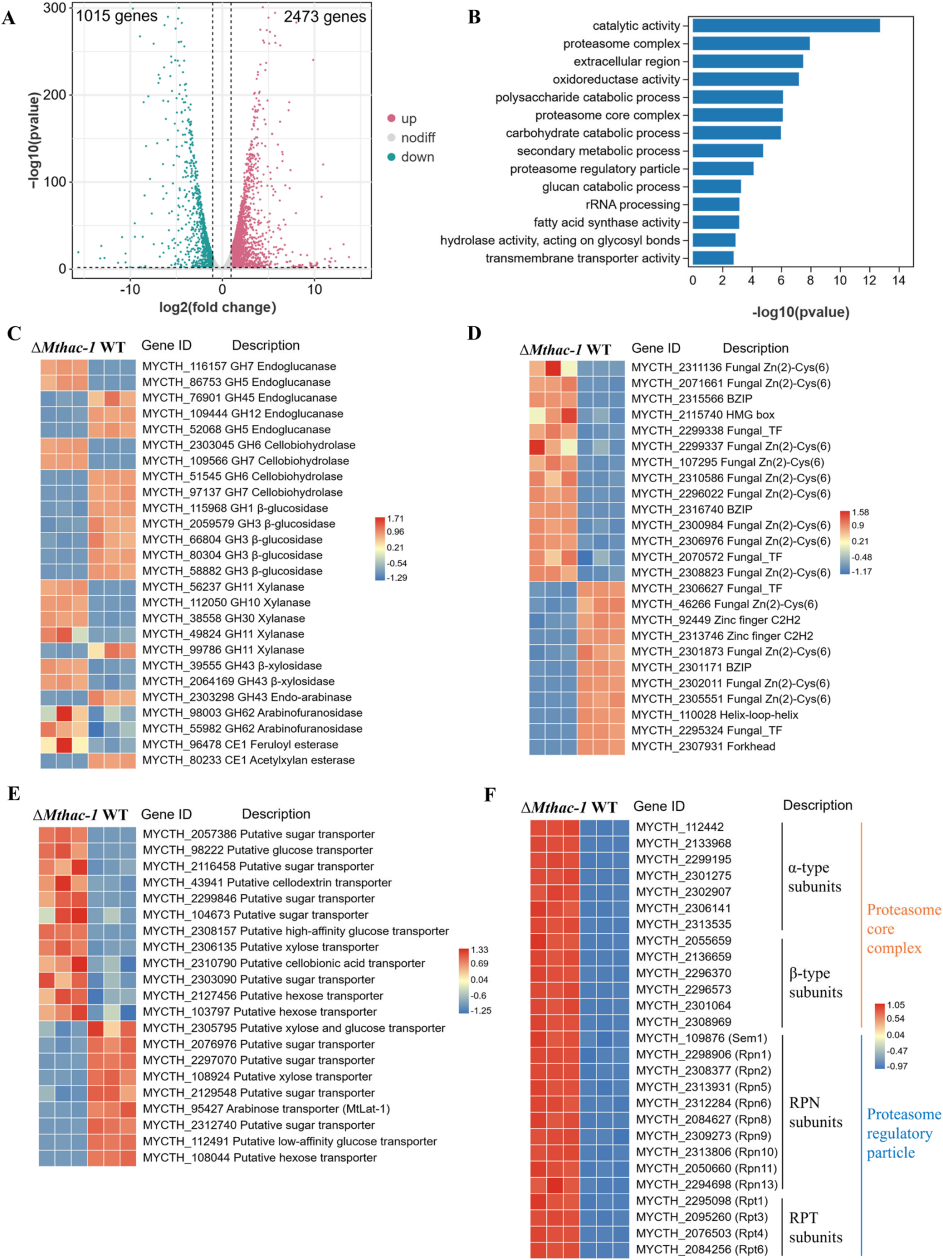
Comparative transcriptomic analysis of strains ΔMthac‐1 and WT cultivated in Avicel medium for 48 h after transfer from glucose medium. (A) Volcano plots illustrating differentially expressed genes (DEGs) between ΔMthac‐1 and WT. The significantly upregulated and downregulated genes in the ΔMthac‐1 strain are plotted in red and green, respectively. (B) Gene Ontology (GO) enrichment analysis of DEGs in the biological process, cellular component, and molecular function categories. (C–F) Heatmap analysis of expression profiles for genes encoding cellulases and xylan‐degrading enzymes (C), putative transcription factors (D), putative sugar transporters (E), and 26S proteasome (F), respectively, between Δ*Mthac‐1* and WT strains.

To comprehensively understand the function of MtHac‐1, comparative transcriptomics of the WT, and Δ*Mthac‐1* strains grown in Avicel medium for 48 h after switching from glucose were assayed by RNA‐Seq. Analysis of differential gene expression uncovered that 3488 genes showed altered transcription levels between the WT and Δ*Mthac‐1* strains, of which 2473 genes were significantly upregulated and 1015 genes were markedly downregulated in the Δ*Mthac‐1* strain (Figure 6A, Table S2). Gene Ontology (GO) analysis of the differentially expressed genes (DEGs) in the Δ*Mthac‐1* mutant indicated that proteasome complex, extracellular region, polysaccharide catabolic process, hydrolase activity (acting on glycosyl bonds), transmembrane transporter activity and so on were significantly enriched functional categories (Figure 6B, Table S3), consistent with the phenotypes observed in Δ*Mthac‐1* under cellulose induction conditions.

Expression pattern analyses of CAZyme genes revealed that those involved in cellulose and xylan degradation were expressed at considerably altered levels (Figure 6C). Two endoglucanase genes were highly induced, and three endoglucanase genes were downregulated in Δ*Mthac‐1*, indicating that *egl2* and *MYCTH_116157* might contribute more to the increased endoglucanase activity. Similarly, the expression of two genes encoding cellobiohydrolase (*cbh1* and *MYCTH_2303045*) was increased and the other two cellobiohydrolase genes were reduced by the absence of *Mthac‐1*. In contrast, transcript levels of five β‐glucosidase genes were significantly decreased in Δ*Mthac‐1* compared to the WT, which was consistent with RT‐qPCR detection showing that *bgl1* displayed a lower expression level in Δ*Mthac‐1* after 48 h of cultivation on Avicel. As expected, many genes encoding xylan‐degrading enzymes exhibited elevated expression levels in Δ*Mthac‐1* relative to the WT on Avicel, including four endo‐xylanases (xyn1, GH11 and GH30), one GH43 β‐xylosidase, and eight xylanolytic accessory enzymes (GH43, GH62, CE1 and ce15), in accordance with the elevated xylanase production in the Δ*Mthac‐1* mutant during the middle and late stages of growth. The increase in expression levels of four LPMO (AA9) genes, which are commonly considered to play an oxidative role in cellulose depolymerisation, was also observed in Δ*Mthac‐1* during 48 h of growth on Avicel (Figure S5). One of these AA9 genes, encoding MtLPMO9C (MYCTH_100518), was reported to be active toward cellulose, implying that the other three LPMO genes regulated by MtHac‐1 might also be associated with the oxidative split of Avicel.

Apart from these CAZyme genes, the genes that encode putative transcription factors also had changed expression levels, which include 14 upregulated and 11 downregulated genes in Δ*Mthac‐1* (Figure 6D). Most of these TFs are classified into Fungal Zn(II)2Cys6 and Fungal TF families. Among these transcriptional regulators, the forkhead protein MtFKH1 (MYCTH_2307931) which was newly identified as a repressor of cellulase and xylanase gene expression (Lai et al. 2025), showed a noticeably lower expression level (log_2_ fold change = −7.2) by the deletion of *Mthac‐1*. This may partly contribute to the enhanced cellulase and xylanase activities. It is worth noting that Mtxyr1, which is a pivotal transcriptional activator of xylanolytic genes, exhibited a 53% increase in the transcript level in the Δ*Mthac‐1*. Despite not meeting the statistical threshold, it still partially agreed with the RT‐qPCR results.

The expression of genes encoding putative sugar transporters was also analysed by comparative transcriptomic analysis. A total of 21 genes, annotated as xylose, glucose, hexose, cellodextrin, cellobionic acid or other putative sugar transporters, showed varying degrees of differential expression (Figure 6E). Twelve of these genes unregulated their transcription in Δ*Mthac‐1* and others reduced compared to the WT. In particular, the transcript level of the gene encoding L‐arabinose transporter MtLat‐1 (MYCTH_95427), which has been recently shown to be involved in the repression of xylanase activity in *M. thermophila* on arabinan but not on xylan (Gu et al. 2023), was decreased (log_2_ fold change = −1.98), indicating that it may also play a role during the presence of cellulose.

A crucial response was also detected in the expression of genes that encode the 26S proteasome (Figure 6F). All the 13 genes encoding the 20S proteasome core complex, including seven α‐type subunits (MYCTH_112442, MYCTH_2133968, MYCTH_2299195, MYCTH_2301275, MYCTH_2302907, MYCTH_2306141, and MYCTH_2313535) and six β‐type subunits (MYCTH_2055659, MYCTH_2136659, MYCTH_2296370, MYCTH_2296573, MYCTH_2301064, and MYCTH_2308969) showed elevated transcript levels in the Δ*Mthac‐1* mutant (Figure 6F). In addition, 14 out of a total of 18 genes encoding the 19S proteasome regulatory particle, including 10 Regulatory Particle Non‐ATPase (RPN) subunits (MYCTH_109876, Sem1; MYCTH_2298906, Rpn1; MYCTH_2308377, Rpn2; MYCTH_2313931, Rpn5; MYCTH_2312284, Rpn6; MYCTH_2084627, Rpn8; MYCTH_2309273, Rpn9; MYCTH_2313806, Rpn10; MYCTH_2050660, Rpn11; and MYCTH_2294698, Rpn13) and four Regulatory Particle ATPase (RPT) subunits (MYCTH_2295098, Rpt1; MYCTH_2095260, Rpt3; MYCTH_2076503, Rpt4; and MYCTH_2084256, Rpt6) were also expressed at higher levels in Δ*Mthac‐1* compared with the WT during growth in Avicel medium (Figure 6F). These data illustrated that MtHac‐1 is closely associated with the protein degradation system, implying its involvement in a complex regulatory network.

## 4 Discussion

Filamentous fungi are known to secrete numerous enzymes in their natural settings to degrade the complex polymetric substances for nutrient uptake (Jadhav et al. 2024). During the cellulolytic response, the nascent lignocellulolytic enzyme polypeptides are translocated into the ER to be folded and processed for secretion (Huberman et al. 2016). This process can result in considerable ER stress and trigger the UPR, which aims to maintain ER homeostasis (Li et al. 2022). In *S. cerevisiae* , the UPR pathway depends on the transcription factor Hac1, which undergoes an unconventional splicing reaction in its mRNA, where a 252‐nucleotide (nt) noncanonical intron is cleaved to generate a translationally active form (Xia 2019). In contrast, in filamentous fungi, such as *T. reesei*, *A. nidulans* , *A. niger,* and *N. crassa* , the loss of a 20‐nt or 23‐nt unconventional intron from the mRNA of HacA/Hac1/Hac‐1 is responsible for mediating the UPR signalling pathway (Saloheimo et al. 2003; Mulder and Nikolaev 2009; Montenegro‐Montero et al. 2015). A similar phenomenon was also observed in *M. thermophila* , where the *Mthac‐1* mRNA undergoes a noncanonical splicing via removing a 23‐nt intron when grown on cellulose, although the amount of unspliced mRNA is more than the spliced form (Li et al. 2022). This indicates that the growth of *M. thermophila* on cellulose solely could trigger the ER stress, which is in line with the *N. crassa* phenotypes in previous studies (Montenegro‐Montero et al. 2015). Structure analysis showed that the *MtHac‐1* ^*u*^ (unspliced) mRNA contains an ORF encoding for 571 amino acids, while the *MtHac‐1* ^*i*^ (unconventional spliced) mRNA encodes a protein of 448 amino acids (Figure 1), which is caused by the removal of this 23‐nt intron, leading to the frameshift in the *M. thermophila hac‐1* mRNA. Furthermore, it has been suggested that the UPR is involved in regulating lignocellulolytic enzyme production (Huberman et al. 2016). For example, a transcriptional down‐regulation mechanism was found in *T. reesei*, where the transcript levels of genes encoding secreted proteins, such as cellulases and xylanases, were reduced in response to ER stress (Pakula et al. 2003). Similarly, the selective transcriptional down‐regulation of the glucoamylase gene has also been observed in *A. niger* upon UPR activation (Al‐Sheikh et al. 2004). In addition, the growth of *N. crassa* on cellulose was severely impaired and no secreted protein was detected in the absence of *hac‐1* (Montenegro‐Montero et al. 2015). Here, we carry out this study to elucidate the role of MtHac‐1 in *M. thermophila* , particularly focusing on its role in lignocellulose degradation. Through a combination of transcriptomic analyses and molecular genetic assays, we showed that MtHac‐1 exerts a dual regulatory effect on cellulase and xylanase production. It directly regulates the expression of key cellulase and xylanase genes while also modulating the transcription of the crucial xylanolytic activator *Mtxyr1*. Transcriptome profiling further revealed that the majority of genes encoding components of the 26S proteasome exhibited increased transcript levels in the Δ*Mthac‐1* mutant, highlighting a tight association between MtHac‐1 and the protein degradation machinery in *M. thermophila* .

It was reported that constitutively activated HacA (the spliced form of *hacA* that lacks the 20‐nt untraditional intron) in *A. niger* triggered the UPR signalling and had a negative effect on the expression of genes encoding starch‐degrading and xylanolytic enzymes, sugar transporters as well as AmyR, a key transcriptional activator involved in starch degradation (Carvalho et al. 2012). In *N. crassa* , however, the loss of *hac‐1* impaired cellulase secretion without affecting transcription levels of cellulase genes in the presence of cellulose (Fan et al. 2015). These findings indicate that MtHac‐1 homologues may play divergent roles in different fungi during the lignocellulolytic response. In this study, the disruption of *Mthac‐1* elevated cellulase, xylanase and extracellular protein production after 72 and 96 h of growth in Avicel medium, whereas the overexpression of *Mthac‐1* decreased both enzymatic activities and protein secretion at the late phase (Figure 2). In contrast, Δ*Mthac‐1* mutants showed reduced cellulase and xylanase activities, as well as decreased protein secretion following 48 h of cultivation on Avicel, contrary to the enhanced levels displayed by the OE‐*Mthac‐1* strain (Figure 2). The discrepancy observed in Δ*Mthac‐1* mutant regarding enzymatic activities and overall protein secretion in the early and middle/late stages of incubation was supported by the RT‐qPCR analysis. These data revealed that the expression levels of key cellulase and xylanase genes in Δ*Mthac‐1* were substantially elevated after 48 and 72 h of induction with Avicel but were strongly down‐regulated after 24 h (except for *xyn1* at 24 h and *bgl1* at 48 h, which will be discussed below) (Figure 4). It appears that MtHAC‐1 may act in a similar manner to that of its homologue *N. crassa* HAC‐1 in the early cultivation period, promoting cellulolytic and xylanolytic enzyme secretion. However, during the middle and later stages, it functions similarly to *A. niger* hacA, exerting a negative effect on cellulase and xylanase production. We propose the dual role of MtHAC‐1 plays in response to cellulose induction: (1) During the early phase of growth on cellulose, *M. thermophila* needs to express and secrete large quantities of enzymes to depolymerise this complex substance for supporting cellular growth (Gu et al. 2023; Liu et al. 2024). This increased demand for protein secretion imposes higher folding and processing requirements, necessitating a fully active and functional UPR to accommodate these enzymes in large amounts (Montenegro‐Montero et al. 2015). In this context, MtHAC‐1 therefore positively regulates lignocellulolytic enzyme secretion. (2) In the middle and late stages of growth, secreted enzymes are sufficient for nutrient acquisition. As a result, the accumulation of large amounts of unfolded or misfolded proteins may occur, triggering a feedback repression mechanism of the UPR signalling, which downregulates the transcription of extracellular enzymes, thereby reducing ER load and preventing excessive energy depletion (Pakula et al. 2003; Al‐Sheikh et al. 2004; Carvalho et al. 2012; Zhang et al. 2020). During this process, MtHAC‐1 represses the secretion of cellulase and xylanase. This dual regulatory behaviour potentially reflects the dynamic role of MtHAC‐1 as a key transcription factor that maintains the balance between enhanced protein secretion and ER processing capacity, ensuring basal growth and cellular homeostasis of *M. thermophila* across different growth phases during the lignocellulolytic response (Montenegro‐Montero et al. 2015; Huberman et al. 2016).

In eukaryotic cells, the 26S proteasome comprises the 20S core complex and the 19S proteasome regulatory particle, which serve to remove proteins that are misfolded or dysfunctional as well as no longer needed, and play fundamentally essential roles in regulating almost all major cellular processes (Mao 2021; Sakata et al. 2021). The expression pattern analysis done in this study showed that the majority of genes encoding the 20S proteasome core complex and 19S proteasome regulatory particle displayed higher transcript levels with the deletion of *Mthac‐1* (Figure 6). Since Mthac‐1 mediates the UPR signalling pathway in *M. thermophila* (Li et al. 2022), it is likely that the deletion of *Mthac‐1* disrupted ER homeostasis, impacted accurate protein folding and the removal of unfolded proteins. The disruption of *Mthac‐1* may trigger the cell's demand for proper degradation of accumulated misfolded proteins, especially during the middle and late cultivation phases in cellulose medium, when cellulase and xylanase secretion are strongly elevated in the Δ*Mthac‐1* mutant. This helps to explain why the enhanced expression levels of genes encoding the 26S proteasome were observed in Δ*Mthac‐1*.

Interestingly, the RT‐qPCR results showed a 3.0‐fold upregulation of *xyn1* in Δ*Mthac‐1* at 24 h under cellulose induction, which conflicted with the reduced xylanase activity detected during the early period. The *M. thermophila* genome encodes at least ten xylanases, classified into GH10 and GH11 families (Karnaouri et al. 2014). Among these, *xyn1* (*MYCTH_112050*) has been reported to exhibit the highest transcript abundance and relatively high secretion levels when grown on various lignocellulosic substrates compared to glucose (Kolbusz et al. 2014; Lai et al. 2025). However, other xylanase genes also show considerable transcript levels and/or secretion capacity in response to lignocellulose induction, such as *xyn2* (*MYCTH_100068*), *xyn3* (*MYCTH_116553*) and *xyn4* (*MYCTH_89603*) (Berka et al. 2011; Kolbusz et al. 2014). The reduced xylanase activity observed in the *ΔMthac‐1* mutant after 48 h of growth in Avicel medium may be attributed to the downregulation of these other xylanase genes at 24 h, despite the upregulation of *xyn1* at that time point. Likewise, RT‐qPCR analysis revealed that *bgl1* was downregulated by 61% in Δ*Mthac‐1* after 48 h of growth in Avicel medium, consistent with transcriptomic data showing reduced transcription of *bgl1* and four other β‐glucosidase genes but was contradictory to the increased β‐glucosidase activity observed at 72 h (Figures 2, 4 and 6). In Δ*Mthac‐1*, the transcript levels of cellulase and xylanase genes after 24, 48 and 72 h of growth on Avicel generally corresponded to the production levels of cellulolytic and xylanolytic enzymes at 48, 72 and 96 h, respectively (Figures 2, 4 and 6). These findings relate to previous observations in the *xpp1* deletion mutant of *T. reesei*, which exhibited higher xylanolytic activity than the parent strain after 72 h, matching the enhanced *xyn2* level after 48 h but not the lower expression at 24 h (Derntl et al. 2015). It appears that the transcript levels of *bgl1* in Δ*Mthac‐1* at 48 and 72 h of cellulose induction correlated with the corresponding β‐glucosidase secretion levels at the same time points. This temporal discrepancy between the transcription and secretion patterns of β‐glucosidase and those of endoglucanase, cellobiohydrolase, and xylanase was probably because of the higher translation/secretion efficiency of β‐glucosidase enzymes, leading to a shorter lag between transcription and secretion. This phenomenon warrants further investigation.

In addition to the major cellulase and xylanase genes, the RT‐qPCR and transcriptomic data presented here also reflected the upregulated expression of regulatory gene *Mtxyr1* in Δ*Mthac‐1* after 48 and 72 h of growth on Avicel (Figure 4). Since MtXyr1 is the pivotal activator of xylanase gene expression, MtHac‐1 was tested to see if it can regulate the expression of xylanase gene directly or indirectly. The EMSA results indicated that MtHac‐1 was capable of binding to the promoter regions of both *Mtxyn1* and *Mtxyr1* (Figure 5), showing a strong preference for the upstream regions of these genes compared to the crucial cellulase genes *Mtbgl1*, *Mtcbh1,* and *Mtegl2*. This study confirmed in vitro interaction between MtHac‐1 and its target genes. However, considering that MtHAC‐1 exhibits distinct temporal roles, possibly due to interactions with co‐regulators, whether such interactions occur in vivo would require further investigation.

Earlier research has shown that Hac1/HacA is necessary for normal growth and development in fungi. In *A. niger* , deletion of *hac‐A* resulted in severe growth defects and almost abolished sporulation on rich media (Mulder and Nikolaev 2009). Also, disruption of *Hac1* has been found to impact hyphal morphology with reduced polarised growth in *Candida albicans* (Wimalasena et al. 2008). Deletion mutants of *hac‐1* in *N. crassa* and *hacA* in *Aspergillus fumigatus* , on the other hand, displayed normal growth in rich solid media, although decreased conidiation was observed in *A. fumigatus* (Richie et al. 2009; Montenegro‐Montero et al. 2015), indicating different roles of MtHac‐1 orthologs in fungal development. In this study, the Δ*Mthac‐1* mutant displayed stunted mycelial growth, and produced less conidia on all tested solid media compared to the WT (Figure 3). A similar growth phenotype was also reported in *A. flavus* , where the loss of *hacA* led to retarded mycelial growth and inhibited conidiation on PDA medium (Yu et al. 2024). The transcriptomic data in the current study revealed that the deletion of *Mthac‐1* downregulated the expression level of *MYCTH_2293942* (log_2_ fold change = −4.5), which encodes the *M. thermophila* ortholog of the membrane flavoprotein TmpA found in *A. nidulans* . An earlier study has reported that TmpA is involved in regulation of asexual reproduction, as the loss of which caused a decreased number of conidia (Soid‐Raggi et al. 2006). Moreover, a forkhead regulatory gene *Mtfkh1*, which controls sporulation in *M. thermophila* (Lai et al. 2025), also showed reduced transcript levels in Δ*Mthac‐1* (log_2_ fold change = −7.2). Therefore, the downregulation of genes encoding MtTmpA and MtFKH1 may contribute to the conidiation defect displayed by the Δ*Mthac‐1* mutant grown on solid media.

In summary, the current study demonstrates that MtHac‐1 plays a dual regulatory role in cellulase and xylanase production, likely maintaining a balance between extracellular enzyme secretion and ER processing capacity. Moreover, MtHac‐1 regulates cellulase and xylanase gene expression through two mechanisms: by directly binding to the promoter regions of major cellulase and xylanase genes with different motifs and modulating the xylanolytic gene activator Mtxyr1. Finally, MtHac‐1 is associated with the 26S proteasome degradation system, and the upregulation of 26S proteasome‐encoding genes in the Δ*Mthac‐1* mutant during the middle stage upon cellulose induction likely represents a compensatory response to the loss of *Mthac‐1*. These findings provide novel insights into the regulatory mechanism of MtHac‐1 in controlling the expression of fungal cellulolytic and xylanolytic genes.

## Author Contributions

**Yapeng Lai:** methodology, data curation, investigation, visualization, writing – original draft, conceptualization. **Juan Wang:** conceptualization, methodology, supervision, funding acquisition. **Ning Xie:** methodology, funding acquisition. **Gang Liu:** conceptualization, methodology, supervision, funding acquisition, writing – review and editing. **Donnabella C. Lacap‐Bugler:** conceptualization, methodology, supervision, writing – review and editing.

## Conflicts of Interest

The authors declare no conflicts of interest.

## Supporting information


**Data S1:** mbt270203‐sup‐0001‐SupplementaryFigures.docx.

**Data S2:** mbt270203‐sup‐0002‐SupplementaryTables.xlsx.

## Data Availability

The data have been submitted to a publicly available repository identified in section 2.6. Transcriptome Analysis and in the Supporting Information: Tables.
