# Supplementary material for: Dual Role of MtHAC‐1 in Regulating Cellulase and Xylanase Production in Myceliophthora thermophila
Source: Microb Biotechnol. 2025 Jul 30;18(8):e70203. doi: 10.1111/1751-7915.70203 (PMC12310822; doi:10.1111/1751-7915.70203)
Supplement: Supplementary file 1 — Data S1: mbt270203‐sup‐0001‐SupplementaryFigures.docx. [file MBT2-18-e70203-s002.docx]

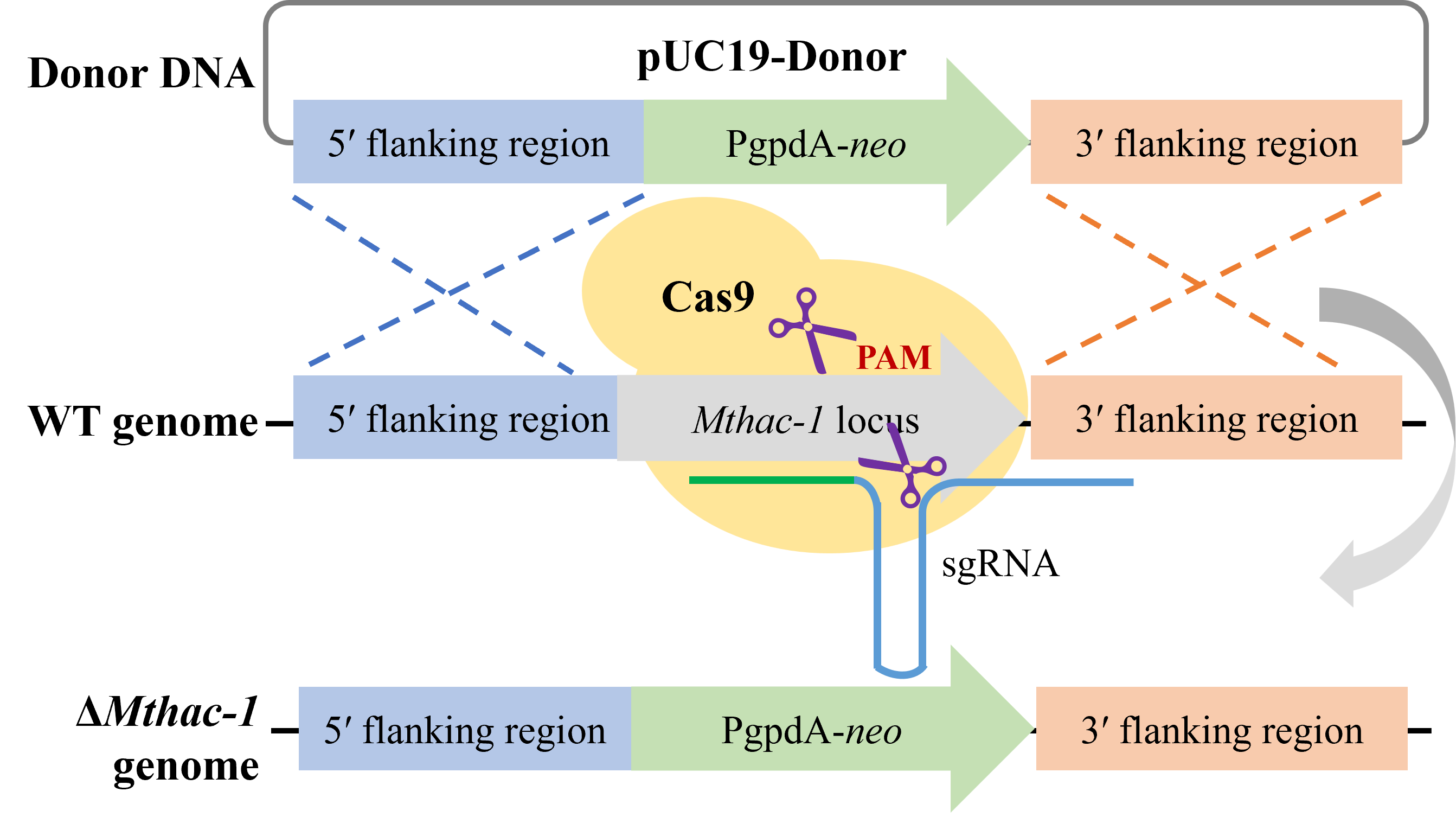


**Figure S1** Schematic representation of the deletion of *Mthac-1* in *M. thermophila* using the CRISPR-Cas9 system. sgRNA, single chimeric guide RNA; PAM, protospacer adjacent motif; *neo*, G418 resistance gene.

**Figure S2** Genomic PCR verification of the deletion and overexpression of *Mthac-1* in *M. thermophila*.


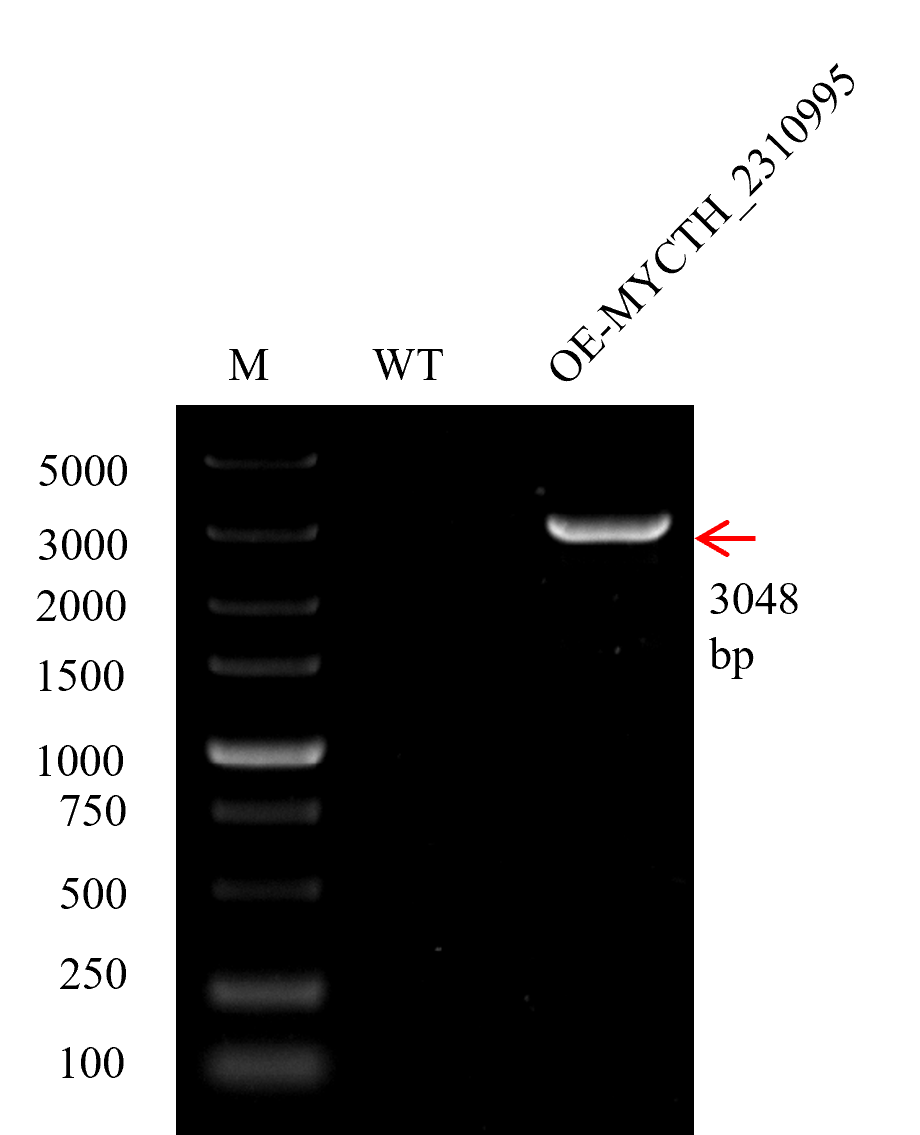


**B**

**A**


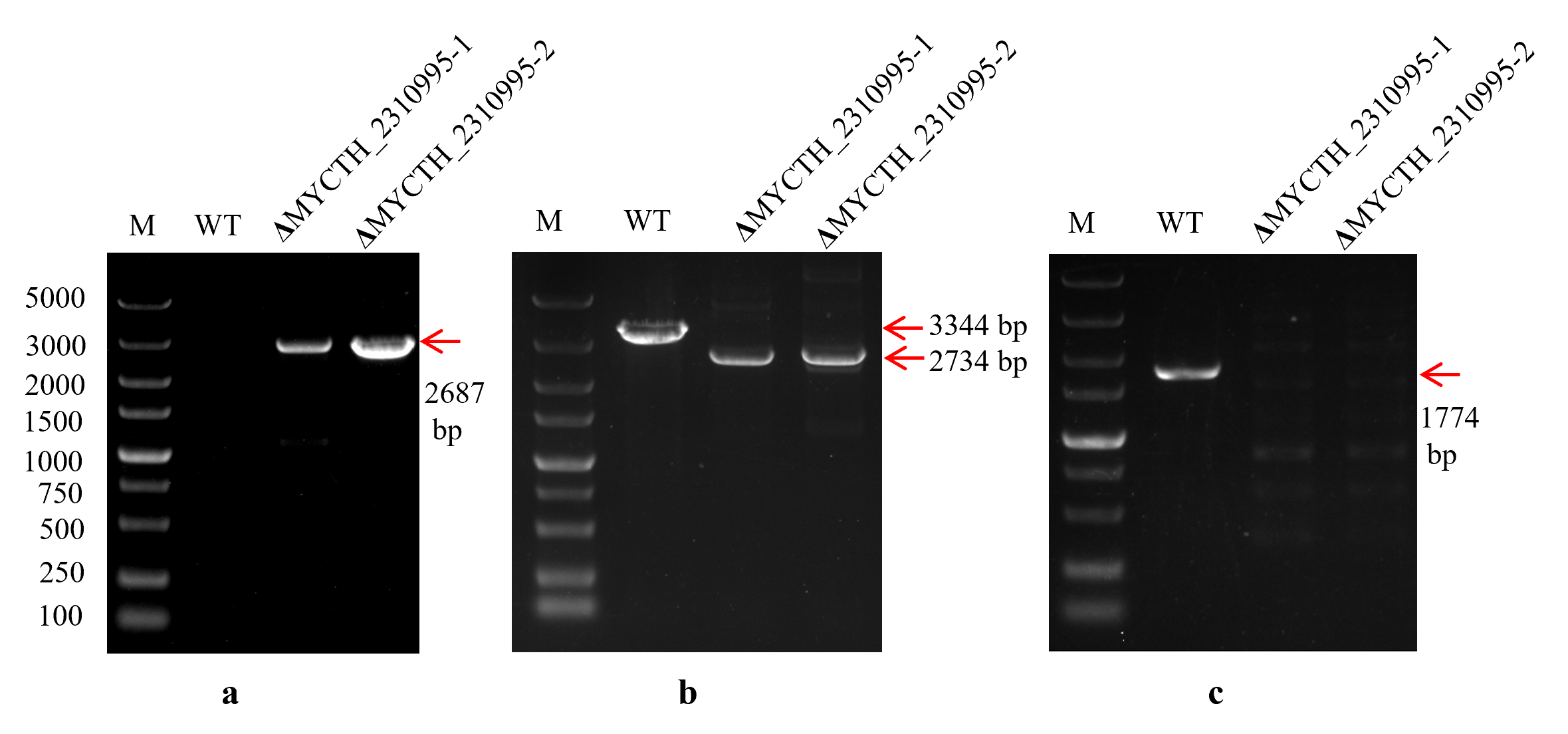


(A) PCR analysis for deletion strains of *Mthac-1*. Primer 1-F locates in P*gpdA*-*neo*, and Primer 1-R locates out of 3′ flanking region of *Mthac-1*; Primer 2-F locates out of 5′ flanking region of *Mthac-1*, and Primer 2-R is in 3′ flanking region; The primer pair 3 is used to amplify *Mthac-1*. a, the primer pair 1; b, the primer pair 2; c, the primer pair 3; M, DL 5000 Marker; WT, M. thermophila wild type strain. (B) PCR analysis for *Mthac-1*overexpression strain. M, DL 5000 Marker; WT, M. thermophila wild type strain.


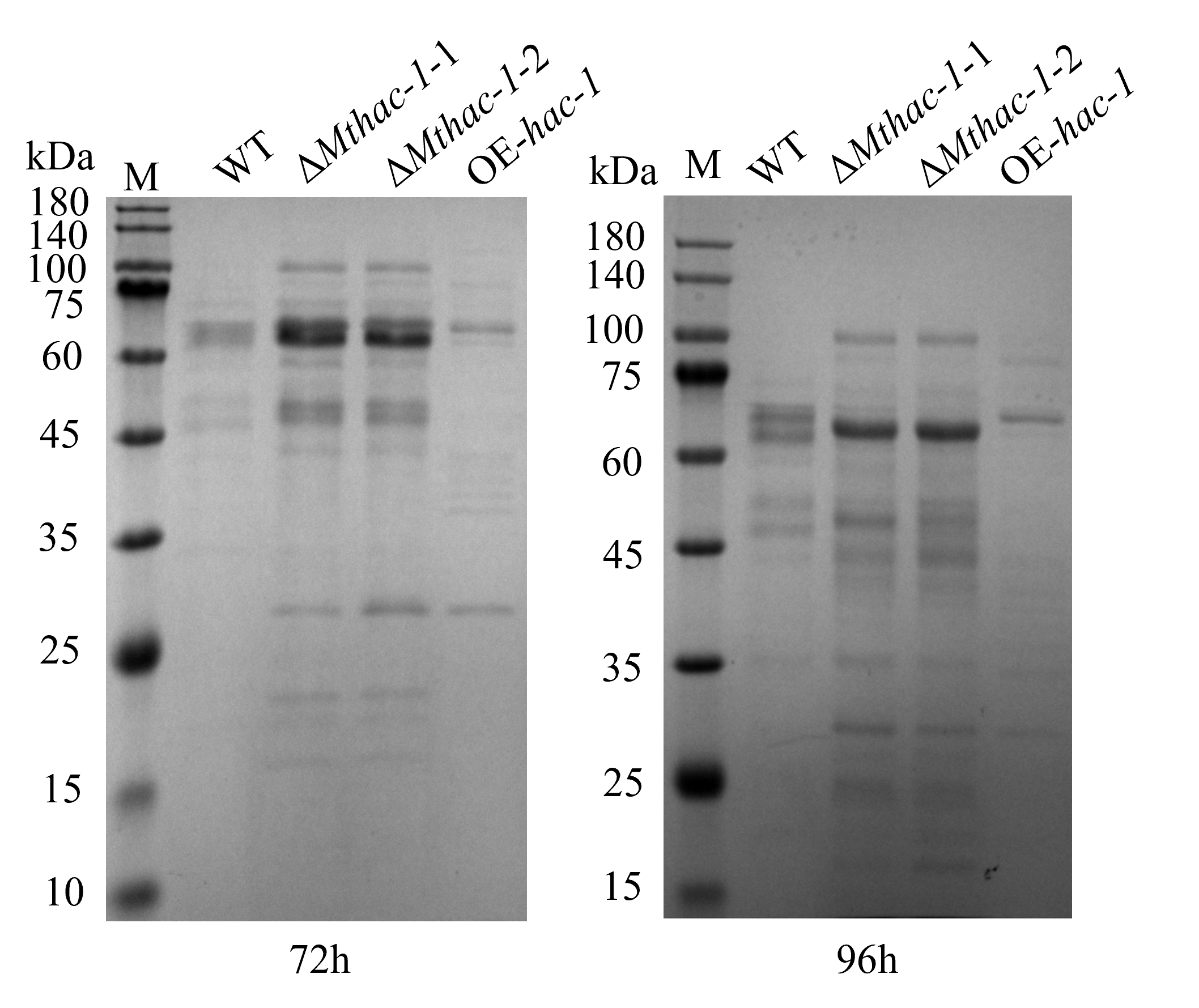


**Figure S3** SDS-PAGE analysis of the secreted proteins in *M. thermophila* WT, Δ*Mthac-1*, and OE-*Mthac-1* strains grown for 72 and 96 h on Avicel after a shift from glucose medium. The protein concentrations of *M. thermophila* WT, Δ*Mthac-1*, and OE-*Mthac-1* strains were 89 µg mL^-1^, 178 µg mL^-1^, and 112 µg mL^-1^, respectively, after 72 h of growth on Avicel. After 96 h of cultivation, the corresponding concentrations were 92 µg mL⁻¹, 238 µg mL⁻¹, and 51 µg mL⁻¹, respectively. M, 10-180 kDa protein marker; WT, *M. thermophila* wild type strain.


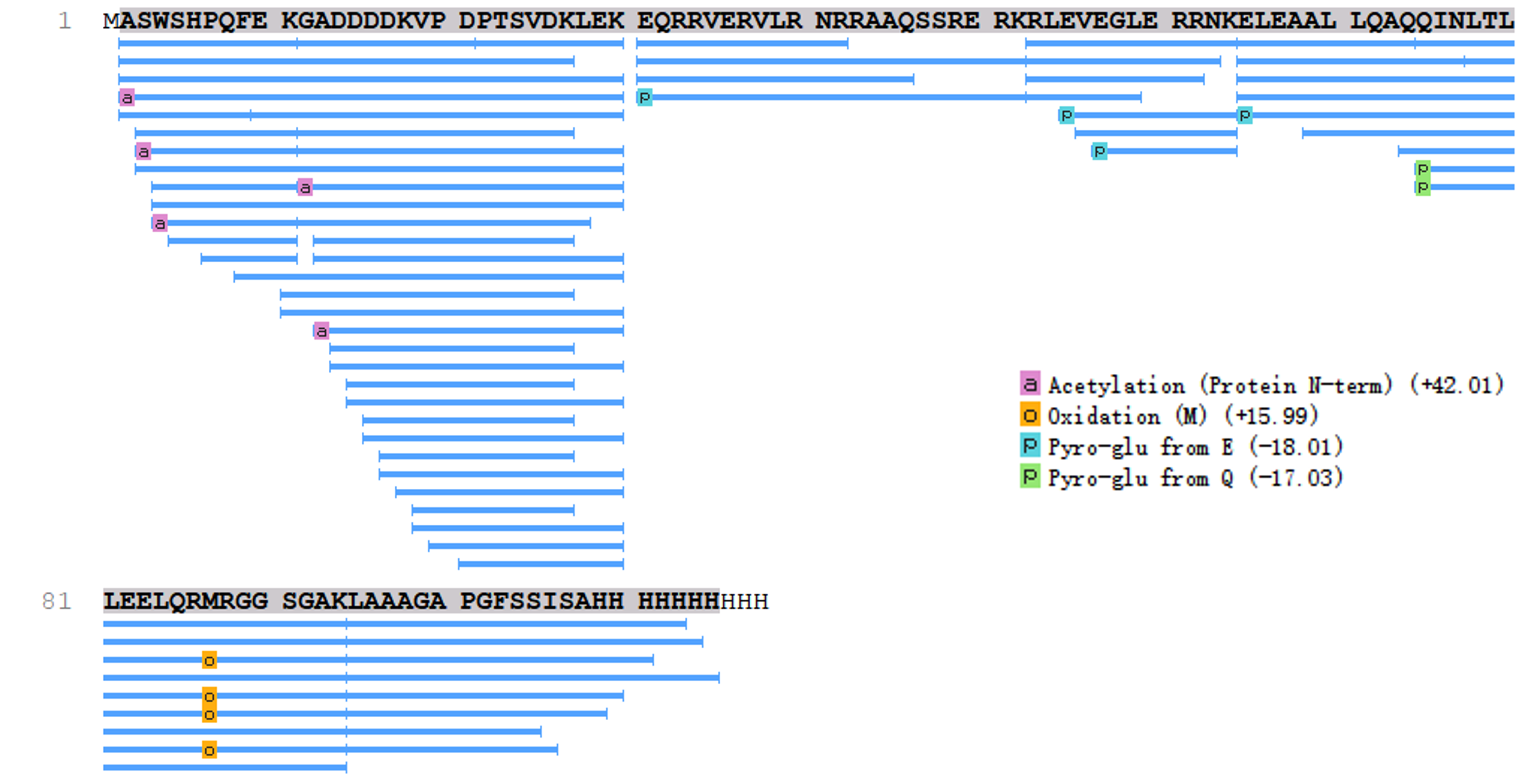


**B**

**A**


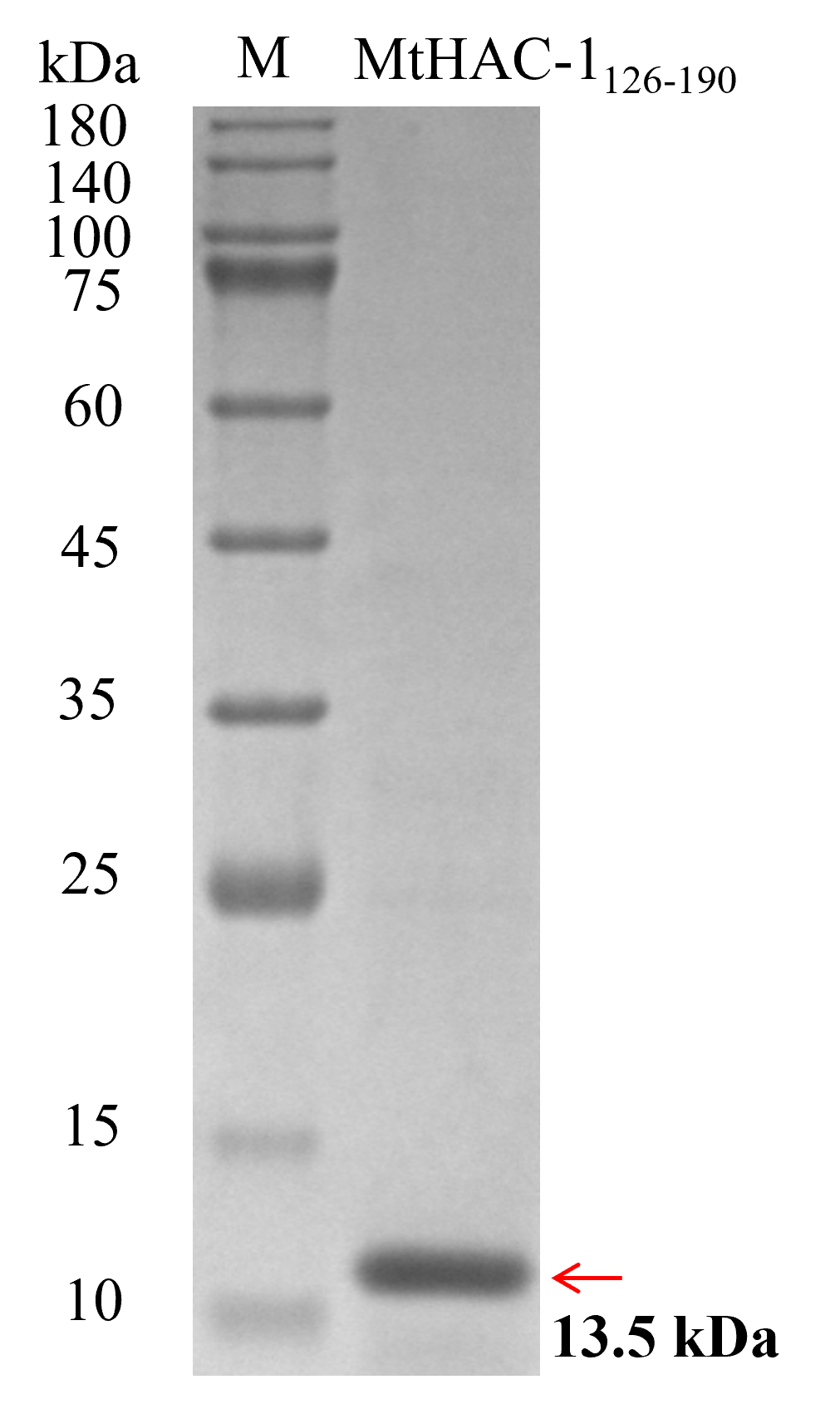


**Figure S4** SDS-PAGE and LC-MS/MS analysis of purified MtHAC-1_126-190_ protein.

(A) SDS-PAGE verification of purified MtHAC-1_126-190_. The sample was loaded onto a 12.5 % polyacrylamide gel. The concentration of the purified MtHAC-1_126-190_ is 398 µg mL^-1^. M, 10-180 kDa. (B) LC-MS/MS characterization of MtHAC-1_126-190_ protein. The target protein band was excised from the gel and analyzed using LC-MS/MS assays with the PEAKS Studio 8.5 system. Approximately 97% of the amino acids of the target protein matched the theoretical amino acid sequence of the Strep II-MtHAC_126-190_-His tag fusion protein.


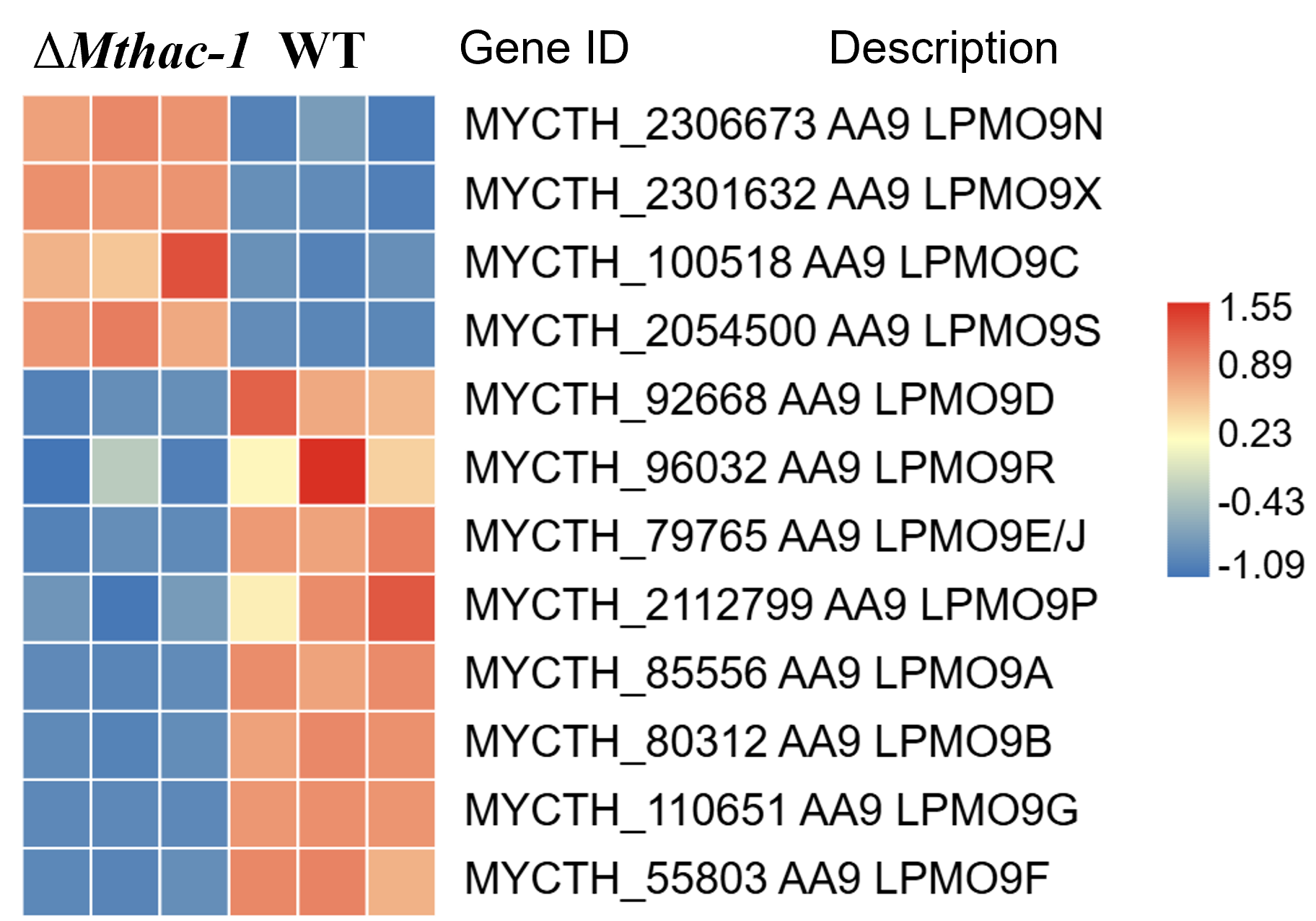


**Figure S5** Heatmap analysis of expression profiles for genes encoding lytic polysaccharide monooxygenases (LPMOs) (AA9) between *M. thermophila* ΔMthac-1 and WT strains grown in Avicel medium for 48 h after a shift from glucose medium.
